# Motor Imagery Practice to Improve Respiratory and Cough Function

**DOI:** 10.1007/s00455-025-10818-2

**Published:** 2025-02-26

**Authors:** Cara Donohue

**Affiliations:** 1 Innovative Research in Aerodigestive Disorders Laboratory, Vanderbilt University Medical Center, Nashville, TN USA; 2 Department of Hearing and Speech Sciences, School of Medicine, Medical Center East Room 10225, Vanderbilt University, 21st Avenue South, Nashville, TN 37232 USA

**Keywords:** Dysphagia, Pulmonary function, Cough, Motor imagery practice, Treatment

## Abstract

Motor imagery practice (MP), or visualizing performing a task without executing it, is an effective intervention for the limbs and tongue. However, MP of expiratory muscle strength training (EMST) and voluntary cough (VC) has not been explored. We examined the feasibility and impact of MP of VC and EMST on pulmonary and cough function in twenty community-dwelling adults. Participants underwent two baseline research evaluations of maximum expiratory and inspiratory pressure (MEP, MIP), forced vital capacity (FVC), voluntary peak expiratory flow rate (PEF), and cough spirometry. After the second research evaluation, participants underwent five weeks of MP of VC or EMST (alternating assignment, 25 repetitions, 70% training load, daily) at home with weekly telehealth sessions. Participants underwent post-treatment research evaluations of MEP, MIP, FVC, PEF, cough spirometry, and the Exercise Therapy Burden Questionnaire (ETBQ). Treatment adherence and telehealth attendance were tracked. Descriptives and Wilcoxon signed rank tests were performed. MP adherence was 95% and telehealth attendance was 91%. Median (IQR) ETBQ scores were 8 (1, 15), indicating minimal burden. Across both MP groups, increases in PEF from a handheld device (+ 13.7, 95% CI: 1.8, 25.6, *p* = *0.03*) and from cough spirometry (+ 0.71, 95% CI: 0.05, 1.4, *p* = *0.04*) were observed. Increases in PEF from cough spirometry (+ 1.00, 95% CI: 0.12, 1.9, *p* = *0.04*) were observed for the MP VC group. Preliminary data demonstrate MP of VC and EMST is feasible and improves voluntary cough in community-dwelling adults. Future research is needed in larger sample sizes and patient populations with pulmonary, cough, and swallowing impairments.

**Supplementary Information:**

The online version contains supplementary material available at 10.1007/s00455-025-10818-2.

## Introduction

Motor imagery practice, which refers to visualizing the performance of a task without executing it, has been investigated as an effective intervention for both physical (e.g., exercises such as squats) and cognitive tasks (e.g., maze solving) [1]. Notably, motor imagery practice has demonstrated efficacy in improving motor performance (i.e., timing and sequencing), motor flexibility, and strength, as well as potential in inducing neuroplasticity due to the overlap between neural representations for motor imagery and physical execution of movements [1, 2]. The impact of motor imagery practice on performance has been explored in musicians, athletes, elderly people, and in patient populations requiring rehabilitation [1, 3–6].

Although the feasibility and efficacy of motor imagery practice has been explored broadly in the limbs, few studies to date have examined the feasibility or efficacy of motor imagery practice to improve respiratory, cough, or swallow function. One recent study examined the impact of motor imagery practice on tongue strength in a case series of healthy adults [7] given that reduced tongue strength is a physiological impairment that can contribute to dysphagia. As such, isometric tongue strengthening exercises have been utilized as one treatment strategy to improve swallow function in patients with dysphagia [8–12]. In this study by Szynkiewicz et al. (2019), six healthy adults underwent motor imagery practice of tongue exercises to improve tongue strength. Individuals completed 30 repetitions of motor imagery practice of tongue exercises, three times daily for three days per week across six weeks. All study participants demonstrated significant pre-to-post motor imagery practice gains in tongue strength [7]. This research study laid the foundation for a randomized controlled trial in 29 healthy older adults which compared active completion of lingual exercises, motor imagery practice of lingual exercises, a combined intervention group, and a control group [13]. Similar to the prior study, individuals in this study completed 30 repetitions, three times daily for three days per week across six weeks, across their respective groups. Interestingly, results revealed the largest gains in tongue strength for the combined intervention group of active lingual exercises and motor imagery practice of lingual exercises (17% vs. 6–9% in other groups). Furthermore, individuals who completed either active lingual exercise or motor imagery practice of lingual exercises demonstrated changes in oxygenation as measured by functional near infrared spectroscopy (fNIRS) post-treatment, which is suggestive of neuroplastic changes [14]. These studies provide preliminary, ‘proof-of-concept’ data regarding motor imagery practice for swallowing exercises, which the current study will expand upon.

Expiratory muscle strength training (EMST) is another commonly utilized dysphagia treatment that aims to improve pulmonary function, cough effectiveness, and swallowing safety [15–26]. The safety and effectiveness of active EMST is well established in healthy adults and in patient populations with dysphagia. Likewise, active interventions targeted at improving voluntary cough function and strength have been safe, feasible, and demonstrated promise in improving voluntary cough function [27–31]. However, no studies to date have explored the feasibility and impact of motor imagery practice of voluntary cough or EMST to determine its feasibility (e.g., viability) and efficacy (e.g., treatment effectiveness in ideal conditions) compared to motor execution of these tasks. Yet, there is data that demonstrates similar brain activation patterns on functional magnetic resonance imaging (fMRI) for motor imagery of voluntary cough and overt voluntary cough in healthy adults [32] as well as for self-feeding and swallowing [33–35], providing support for the current study which explored motor imagery practice for EMST and voluntary cough. Notably, motor imagery practice may be particularly useful as an intervention or adjuvant treatment in patient populations that are weak or deconditioned (e.g., frail individuals, geriatric adults, acutely ill individuals), fatigue easily with exercise (e.g., neurodegenerative disease patient populations), who may benefit from an intervention targeting neuroplasticity/motor performance in addition to muscle strength, and who may have contraindications to active exercise performance (e.g., postoperative patients).

Therefore, the current study aimed to determine (1) the feasibility (e.g., viability) of motor imagery practice of voluntary cough and EMST, and (2) the efficacy (e.g., treatment effectiveness in a controlled study) of motor imagery practice to improve voluntary cough and EMST on physiologic metrics of pulmonary and cough function in a cohort of community-dwelling adults. We hypothesized that motor imagery practice of voluntary cough and EMST would be feasible as determined by high treatment adherence and telehealth session attendance (> 90%) and that it would be efficacious as demonstrated by improvements in maximum expiratory pressure (MEP), voluntary peak expiratory flow rate (PEF), and voluntary cough spirometry metrics.

## Methods

### Research Participants

This study was approved by our University’s Institutional Review Board (#230870) and all research participants provided written informed consent. Inclusion criteria for study enrollment was as follows based on participant self-report: (1) community-dwelling adult between the ages of 18–60 years old, (2) no history of respiratory disease, (3) no history of neurological disease, (4) no history of head and neck cancer or surgery to the head and neck region, (5) no history of swallowing difficulties, and (6) not currently pregnant.

### Equipment and Procedures

#### Study Design

**Fig. 1:**
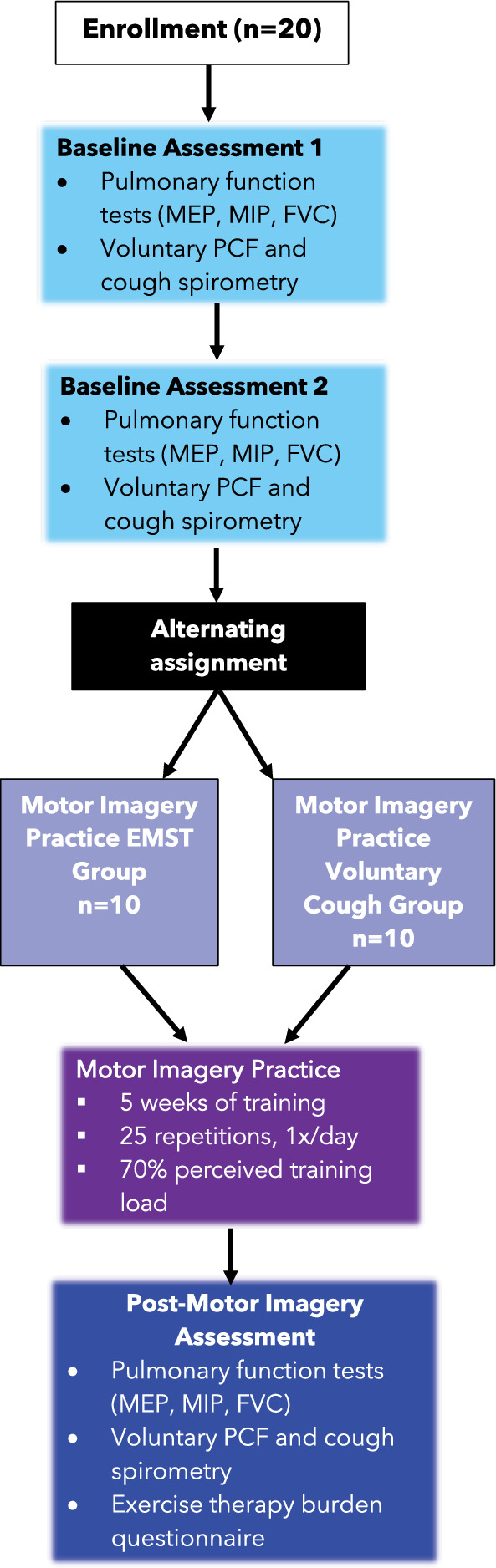
Flowchart of study design and procedures

This was a prospective cohort research study involving two motor imagery practice treatment groups: voluntary cough and EMST. Research participants underwent three separate research evaluations consisting of standardized pulmonary and cough function testing during this study (1) Two evaluations prior to commencing the motor imagery practice program (e.g., multiple baseline to account for a learning effect), (2) One evaluation after completing five weeks of a motor imagery practice program of voluntary cough or EMST. The methods for the research evaluations are outlined in detail below and in Fig. 1.

#### Motor Imagery Practice Training Protocol

Enrolled research participants underwent five weeks of motor imagery practice of either voluntary cough or EMST following completion of research evaluations one and two (multiple baseline). The second baseline visit was conducted approximately one week after the initial visit to account for a learning effect. No mental practice was performed between research evaluations one and two. The initial treatment session for motor imagery practice took place in our research laboratory on the same day as the second baseline research evaluation. Research participants were alternately assigned to the motor imagery practice voluntary cough or motor imagery practice EMST treatment group. After the initial visit, the motor imagery practice program was completed from research participant’s homes using a counter clicker. The counter clicker was used to assist participants in tracking the number of repetitions that they had completed. For completion of motor imagery practice repetitions, research participants were trained to imagine 70% of their voluntary peak expiratory flow rate (PEF) or maximum expiratory pressure (MEP) resistance. Given that all pulmonary and cough function tests obtained during research visits were performed at maximal effort (100%), an initial active training session was performed at 70% effort. To do this, research participants were given a limited number of active trials targeting 70% effort (± 10%) using a manometer device (MEP, EMST group) or an analog peak cough flow meter (PCF, voluntary cough group). Participants demonstrated proficiency in performing active EMST or voluntary cough at 70% effort prior to leaving the laboratory. This was measured by participants’ ability to target 70% (± 10%) resistance on 8/10 trials. After successfully completing active trials at 70% resistance, participants were provided with instructions for performing motor imagery practice of EMST or voluntary cough. Participants demonstrated proficiency in performing motor imagery practice of EMST or voluntary cough at 70% imagined effort prior to leaving the laboratory. This was measured by execution of at least five motor imagery repetitions of EMST or voluntary cough without extraneous observable movement of the head, neck, or trunk muscles involved in respiratory and cough function. The first complete treatment session was conducted the next day via telehealth to reinforce learning. Additionally, participants were provided a training manual with detailed instructions to provide reinforcement of the initial training session (See supplementary materials, adapted) [7]. The motor imagery practice training protocol involved training seven days per week and performing five sets of five repetitions (i.e., 25 repetitions). Participants recorded their daily treatment sessions and repetitions via MyCap [36], a secure research participant phone application, to track adherence and had weekly telehealth sessions with a trained research assistant via a secure version of Zoom (Zoom, San Jose, CA) to ensure quality of repetitions. Given that motor imagery practice involves visualization of executing a movement without executing a movement, research assistants ensured quality of motor imagery practice of repetitions by observing research participants for any overt movements or muscular activation of the head, neck, and trunk muscles that are involved in respiratory and cough function beyond normal resting breathing as has been reported in other preliminary motor imagery practice studies [7]. This included any movements that would be associated with active execution of EMST or voluntary cough such as lip rounding, opening the mouth, activation of the submental muscles, voicing, throat clearing, or breathing in and out forcefully. Treatment adherence and telehealth session attendance were used to determine treatment feasibility. Research participants then underwent a third research evaluation in our laboratory after completing five weeks of motor imagery practice of either EMST or voluntary cough.

### Research Evaluation Measures

#### Maximum Expiratory Pressure, Maximum Inspiratory Pressure, Forced Vital Capacity

Pulmonary function measures were obtained by a trained research assistant in accordance with standardized protocols within our research laboratory and in line with guidance provided by the American Thoracic Society [37–39]. Measurements were obtained with research participants in an upright seated position with nose clips in place. For MEP and MIP pulmonary function measures, three measurements were obtained, and the highest value was utilized for subsequent analyses. The MicroRPM handheld device (Micro Direct Inc., Lewiston, ME) was used to obtain measurements of MEP and MIP. For MEP measurements, research participants were instructed to take a deep breath in, place their mouth around the mouthpiece, and exhale as hard and fast as possible against the resistance. As needed, a research assistant aided participants in obtaining adequate lip seal during MEP measurements by holding the sides of participants’ cheeks. For MIP measurements, research participants were instructed to breathe all the air out of their lungs, place their mouth around the mouthpiece, and inhale as hard and fast as possible against the resistance. A handheld spirometer (Micro I; Carefusion,Yorba Linda, California) was used for forced vital capacity (FVC) measurements. For FVC pulmonary function measures, the two highest measurements differed ≤ 0.150 L, and the highest FVC obtained was utilized for subsequent analyses. For FVC testing, research participants were instructed to take a deep breath in, place their mouth around the mouthpiece, and to blow all the air out of their lungs for as long as possible.

#### Voluntary Cough Peak Expiratory Flow (PCF) and Cough Spirometry

Cough function measures were obtained by a trained research assistant in accordance with standardized protocols within our research laboratory and were obtained with research participants in an upright seated position with nose clips in place. Three measurements of a single cough were obtained for voluntary PEF and cough spirometry, and the highest value was used for subsequent analyses. A handheld analogue Mini-Wright peak flow meter (Clement Clarke Int., Harlow, United Kingdom) with a disposable one-way mouthpiece was used for voluntary PEF measurements. For voluntary PEF, research participants were instructed to take a deep breath in, place their mouth around the mouthpiece, and to cough hard like something was stuck in their throat. An oral pneumotachograph (MLT 1000; ADInstruments, Inc., Colorado Springs, Colorado) attached to a PowerLab C with a spirometer (ADInstruments, Colorado Springs, CO) was used for voluntary cough spirometry measurements and LabChart Version 8 (Microsoft Corp., Redmond, Washington) was used to record cough waveforms on a MacBook Air laptop computer for subsequent analyses. For voluntary cough spirometry, research participants were instructed to breathe normally for several breath cycles prior to taking a deep breath in and coughing hard like something was stuck in their throat. We obtained measures of voluntary cough in two different manners due to their respective strengths and limitations. Voluntary PEF with a handheld meter is more easily deployable within clinical settings. While less readily available within clinical settings, voluntary cough spirometry with a pneumotachograph is considered the gold standard for obtaining quantitative cough measures. Given that there is conflicting evidence regarding concordance between handheld analog peak cough flow devices and voluntary cough spirometry in the literature, we elected to include both measures in this study [40].

#### Exercise Therapy Burden Questionnaire

During the final research evaluation visit, research participants also completed the Exercise Therapy Burden Questionnaire (ETBQ) [41] on an iPad. The ETBQ is a validated 10-item questionnaire. Individuals rate each statement from 0 (totally disagree) to 10 (totally agree). As such, scores on the ETBQ can range from 0–100 and higher scores indicate a greater degree of burden.

#### Voluntary Cough Spirometry Analysis

Two trained raters performed voluntary cough spirometry measurements of inspiratory phase duration, inspiratory peak flow rate, compression phase duration, expiratory phase duration, peak expiratory flow rate, and cough volume acceleration in a blinded and randomized manner [40]. The primary rater performed all voluntary cough spirometry measurements. To assess intra-rater reliability of measurements, the primary rater randomly selected 20% of cough spirometry files to re-rate at least two weeks after the initial ratings were completed. To assess inter-rater reliability, the secondary rater randomly selected 20% of cough spirometry files to perform voluntary cough spirometry measurements on. Intra-class correlation coefficients (ICCs) with 95% confidence intervals (CI) were used to calculate inter and intra-rater reliability for voluntary cough spirometry metrics. Inter and intra-rater reliability for voluntary cough spirometry metrics were excellent (0.92, 95% CI: 0.89, 0.94) and (0.96, 95% CI: 0.94, 0.97), respectively.

### Statistical Analysis

No prior studies examining motor imagery practice of EMST or voluntary cough have been conducted, so an a priori power analysis was conducted in G*Power 3.1 [42] using a medium effect size based on prior research studies examining active EMST in healthy adults. The power analysis revealed that a total sample of 14 community-dwelling adults was needed for a desired power of 0.80 (Type I error rate of 0.05) and a predicted power of 0.82 for N = 14 participants. Based on this, we determined that a sample size of 20 participants would provide adequate preliminary data of motor imagery practice of EMST and voluntary cough.

All study data were entered into our secure online database, REDCap and the associated application, MyCap [36, 43, 44]. After data collection was complete, data were exported from REDCap and MyCap into JMP Pro version 17.0.0 [45] and GraphPad Prism Version 10.3.1 to conduct statistical analyses and make figures. The highest value obtained at each timepoint for physiologic metrics of pulmonary and cough function were used for analyses given that these are maximum performance tasks. Descriptive statistics (frequency/percentage, mean/standard deviation, median/interquartile range) were used to summarize participant demographics, treatment adherence, telehealth session attendance, ETBQ scores, and pulmonary and cough function outcome measures across timepoints. Individual participant and group level spaghetti plots were created to demonstrate individual variability and group-level variability across timepoints and outcome measures. The significance level for statistical analyses were set at 0.05. Mean differences with 95% confidence intervals (CIs) and Wilcoxon signed rank tests were used to evaluate change in physiologic metrics of pulmonary and cough function across timepoints for both groups, the motor imagery practice EMST group, and the motor imagery practice voluntary cough group (pre-motor imagery practice vs. post-motor imagery practice). The baseline two visit was used for the pre-motor imagery practice timepoint given the potential learning effect between the two baseline visits.

## Results

Twenty healthy community-dwelling adults met the inclusion criteria and enrolled in this study. One individual withdrew from the study after the first baseline visit due to reasons unrelated to the study. Demographic information for research participants who completed the study is summarized in Table 1.

**Table 1:** Research participant demographics

Participant characteristics (N = 19)	Mean (SD)
Age (years)	33.8 ± 10.4
	Frequency (%)
Sex	
Male	3 (15.8%)
Female	16 (84.2%)
Race	
White	16 (84.2%)
Black	1 (5.3%)
Asian	1 (5.3%)
More than one race	1 (5.3%)
Ethnicity	
Not Hispanic	18 (94.7%)
Hispanic	1 (5.3%)

Proficiency in performing active EMST or voluntary cough at 70% effort during the initial training session was 84% (range 80–100% accuracy across 10 trials). Adherence for the motor imagery practice program was 95% (14,206/15,125 completed repetitions) and telehealth session attendance was 91% (71/78 sessions) across research participants. Research participants did not report any negative side effects from completing the motor imagery practice program of voluntary cough or EMST. Across both groups, median (IQR) for ETBQ scores were 8 (1, 15), indicating minimal burden. The items rated as having the most burden included “I get bored when I exercise (too much repetition, not enough fun)” and “I lack motivation to exercise.” A complete summary of ETBQ scores for each MP group are summarized in Tables 2 and 3, respectively.

**Table 2:** Summary of the median and interquartile range (IQR) of the Exercise Therapy Burden Questionnaire (ETBQ) scores after completing motor imagery practice of expiratory muscle strength training (EMST)

Median (IQR) of ETBQ scores post-EMST MP	
(1) The exercises cause me pain	0 (0, 0)
(2) The exercises cause me fatigue	0 (0, 1)
(3) I get bored when I exercise (too much repetition, not enough fun)	2 (0.5, 6.5)
(4) The exercises in my program are too difficult	0 (0, 0)
(5) I waste too much time exercising	0 (0, 0.5)
(6) Exercising reminds me of my condition	0 (0, 2)
(7) I lack support to exercise	0 (0, 0)
(8) I lack motivation to exercise	2 (0, 3.5)
(9) My exercises are not adapted to my physical activity objectives	0 (0, 0)
(10) I feel that exercising is not efficient in my case	0 (0, 1)
ETBQ total score:	6 (1.5, 14)

**Table 3:** Summary of the median and interquartile range (IQR) of the Exercise Therapy Burden Questionnaire (ETBQ) scores after completing motor imagery practice (MP) of voluntary cough (VC)

Median (IQR) of ETBQ scores post-voluntary cough MP	
(11) The exercises cause me pain	0 (0, 0)
(12) The exercises cause me fatigue	0 (0, 0)
(13) I get bored when I exercise (too much repetition, not enough fun)	3 (0, 5.25)
(14) The exercises in my program are too difficult	0 (0, 0)
(15) I waste too much time exercising	0 (0, 0)
(16) Exercising reminds me of my condition	0 (0, 0.75)
(17) I lack support to exercise	0 (0, 0)
(18) I lack motivation to exercise	2 (0, 5)
(19) My exercises are not adapted to my physical activity objectives	0 (0, 1.25)
(20) I feel that exercising is not efficient in my case	0 (0, 5)
ETBQ total score:	9 (0.75, 17)

### Combined Motor Imagery Practice of EMST and Voluntary Cough Findings

**Fig. 2:**
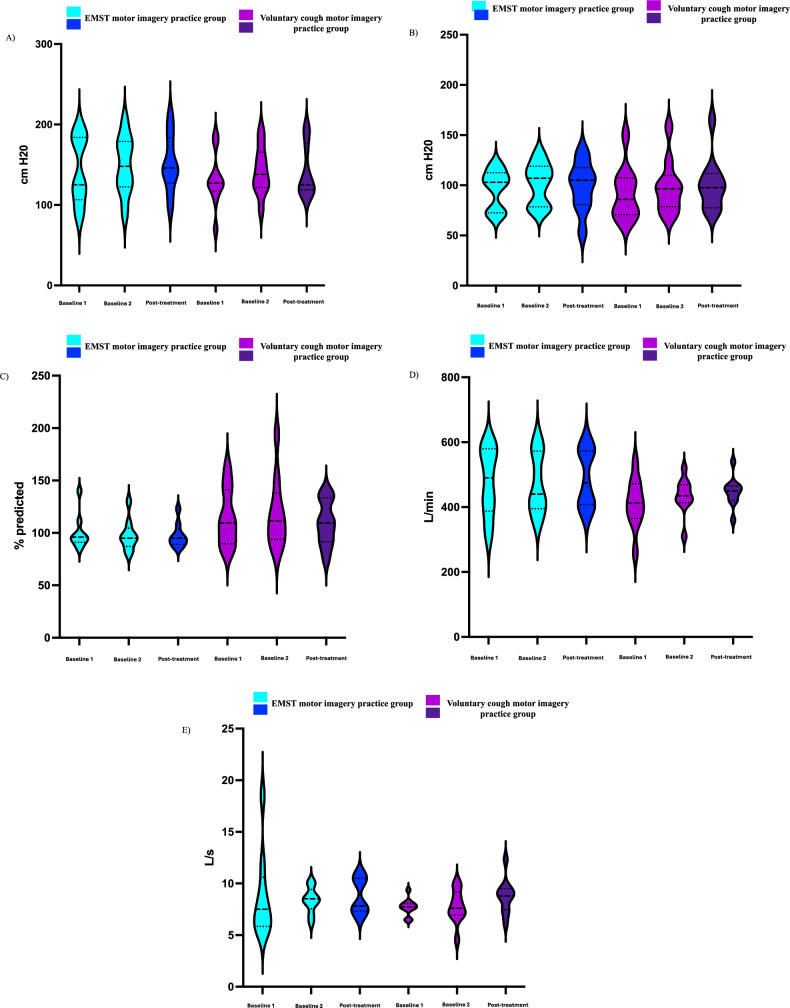
Violin plots for the expiratory muscle strength training and voluntary cough motor imagery practice groups across baseline two and post-treatment time points for **A** maximum expiratory pressure (cm H_2_0), **B** maximum inspiratory pressure (cm H_2_0), **C** forced vital capacity (% predicted), **D** handheld voluntary peak expiratory flow rate (L/minute), and **E** cough spirometry peak expiratory flow rate (L/second)

Descriptive information for pulmonary and cough function measures for both motor imagery practice groups is summarized in Table 4 and Fig. 2. Across both motor imagery practice groups, significant increases in PEF from a handheld cough device (+ 13.7, 95% CI: 1.8, 25.6, *p* = *0.03*) and PEF from cough spirometry (+ 0.71, 95% CI: 0.05, 1.4, *p* = *0.04*) were noted. No other statistically significant increases were observed across both motor imagery practice groups.

**Table 4:** Summary of the median and interquartile range (IQR) of pulmonary and cough function outcome measures during baseline and post-motor imagery practice for both groups

Outcome measures	Median (IQR) baseline 1	Median (IQR) baseline 2	Median (IQR) post- MP	Mean difference (95% CI)	P-value
Pulmonary function tests					
Maximum expiratory pressure (cm H20)	127 (120, 179)	140 (122, 178)	139 (119, 174)	0.68 (− 6.2, 7.6)	0.91
Maximum inspiratory pressure (cm H20)	99 (72, 108)	98 (80, 116)	100 (78, 111)	0 (− 4.6, 2.2)	0.76
Forced vital capacity (% predicted)	99 (91, 119)	99 (90, 120)	99 (91, 115)	− 5.1 (− 11.9, 1.8)	0.04*
Voluntary cough peak expiratory flow and cough spirometry metrics					
Peak expiratory flow rate from a handheld device (L/min)	430 (370, 540)	440 (410, 500)	450 (420, 540)	13.7 (1.8, 25.6)	0.03*
Inspiratory phase duration (s)	1.7 (1.2, 2.7)	1.3 (1.2, 2.3)	1.6 (1.3, 2.6)	0.4 (− 0.1, 0.0)	0.16
Inspiratory peak flow rate (L/s)	− 1.9 (− 2.4, − 1.3)	− 2.1 (− 3.3, − 1.6)	− 2.1 (− 2.8, − 1.6)	0.04 (− 0.5, 0.5)	0.95
Compression phase duration (s)	0.2 (0.1, 0.2)	0.2 (0.1, 0.2)	0.2 (0.1, 0.2)	− 0.03 (− 0.1, 0.07)	0.84
Expiratory phase duration (s)	0.04 (0.03, 0.04)	0.04 (0.03, 0.04)	0.04 (0.03, 0.04)	0.006 (− 0.003, 0.02)	0.31
Peak expiratory flow rate from cough spirometry (L/s)	7.7 (6.5, 8.2)	8.1 (6.9, 8.9)	8.7 (7.6, 10.1)	0.71 (0.05, 1.4)	0.04*
Cough volume acceleration (L/s/s)	227.5 (166.8, 240.1)	223.1 (176.5, 269.9)	232.6 (166.8, 294.9)	− 3.71 (− 51.9, 44.5)	0.68

### Motor Imagery Practice Data for EMST Group Only

**Fig. 3:**
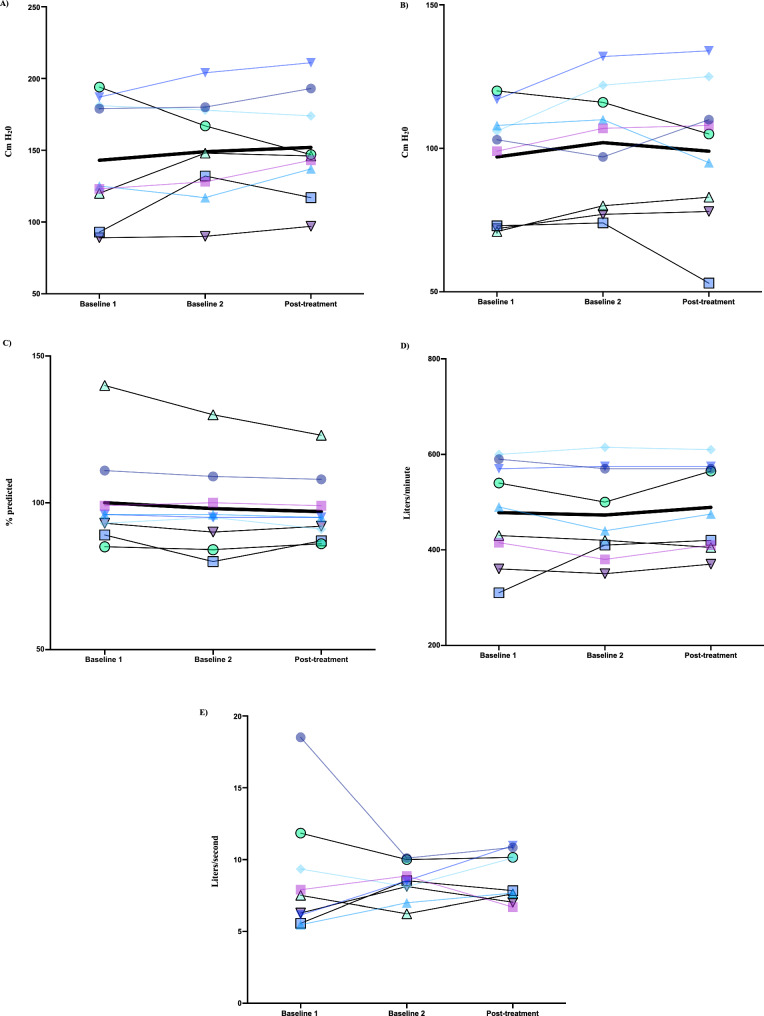
Mean change in pulmonary and cough function measures for the expiratory muscle strength training motor imagery practice group across baseline and post-treatment time points for **A** maximum expiratory pressure (cm H_2_0), **B** maximum inspiratory pressure (cm H_2_0), **C** forced vital capacity (% predicted), **D** handheld voluntary peak expiratory flow rate (L/minute), and **E** cough spirometry peak expiratory flow rate (L/second)

Descriptive information for pulmonary and cough function measures for the EMST motor imagery practice group is summarized in Table 5 and Fig. 3. No statistically significant increases were observed for the EMST motor imagery group.

**Table 5:** Summary of the median and interquartile range (IQR) of pulmonary and cough function outcome measures during baseline and post-motor imagery practice for the expiratory muscle strength training (EMST) group

Outcome measures	Median (IQR) baseline 1	Median (IQR) baseline 2	Median (IQR) Post-EMST MP	Mean difference (95% CI)	P-value
Pulmonary function tests					
Maximum expiratory pressure (cm H20)	125 (107, 184)	148 (123, 179)	146 (127, 184)	2.3 (− 8.1, 12.8)	0.63
Maximum inspiratory pressure (cm H20)	103 (73, 113)	107 (79, 119)	105 (81, 118)	− 2.7 (− 10.9, 5.6)	0.98
Forced vital capacity (% predicted)	96 (91, 105)	95 (87, 105)	95 (89, 104)	− 0.3 (− 3.4, 2.7)	0.88
Voluntary cough peak expiratory flow and cough spirometry metrics					
Peak expiratory flow rate from a handheld device (L/min)	490 (388, 580)	440 (395, 573)	475 (408, 573)	15.6 (− 3.5, 34.6)	0.11
Inspiratory phase duration (s)	1.8 (1.2, 3.0)	1.3 (0.9, 2.9)	1.6 (1.4, 2.8)	0.3 (− 0.2, 0.9)	0.25
Inspiratory peak flow rate (L/s)	− 1.9 (− 2.8, − 1.3)	− 2.4 (− 3.2, − 1.8)	− 1.9 (− 2.7, − 1.5)	0.59 (− 0.2, 1.3)	0.20
Compression phase duration (s)	0.2 (0.1, 0.3)	0.2 (0.1, 0.3)	0.2 (0.1, 0.2)	− 0.03 (− 0.1, 0.1)	0.73
Expiratory phase duration (s)	0.04 (0.03, 0.04)	0.04 (0.03, 0.04)	0.04 (0.03, 0.06)	0.01 (− 0.01, 0.03)	0.52
Peak expiratory flow rate from cough spirometry (L/s)	7.5 (5.9, 10.6)	8.5 (7.5, 9.4)	7.8 (7.3, 10.5)	0.4 (− 0.8, 1.5)	0.50
Cough volume acceleration (L/s/s)	183.6 (157.3, 403.5)	219.3 (192.3, 315.5)	200.9 (129.0, 319.6)	− 41.0 (− 134.8, 52.8)	0.57

### Motor Imagery Practice Data for Voluntary Cough Group Only

**Fig. 4:**
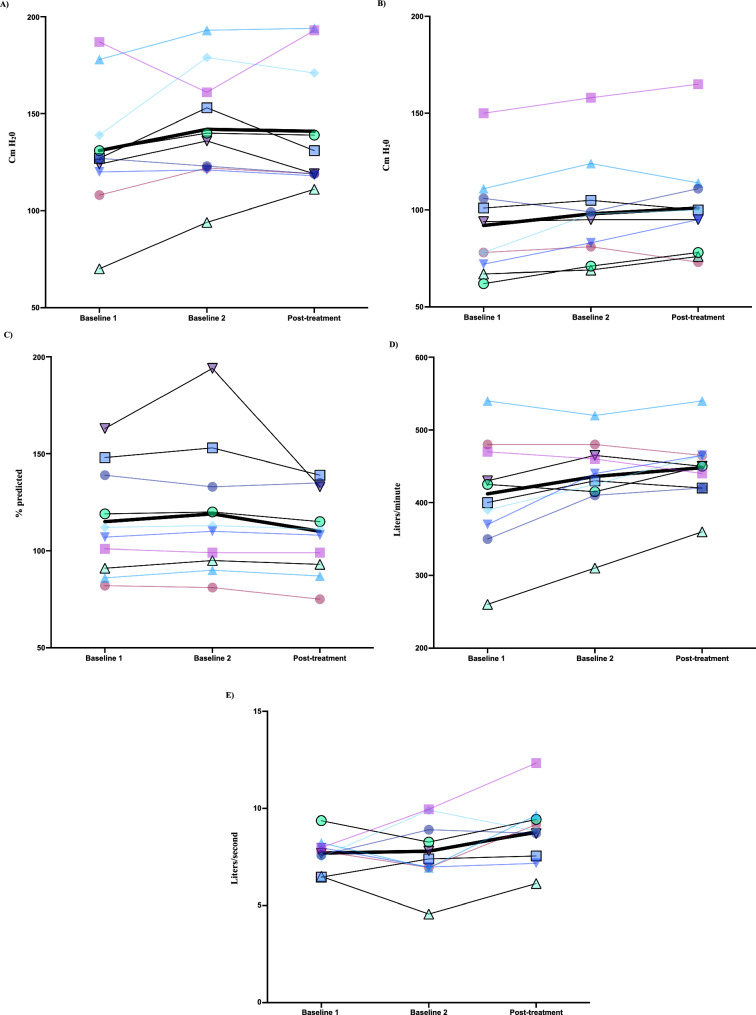
Mean change in pulmonary and cough function measures for the voluntary cough motor imagery practice group across baseline and post-treatment time points for **A** maximum expiratory pressure (cm H_2_0), **B** maximum inspiratory pressure (cm H_2_0), **C** forced vital capacity (% predicted), **D** handheld voluntary peak expiratory flow rate (L/minute), and **E** cough spirometry peak expiratory flow rate (L/second)

Descriptive information for pulmonary and cough function measures for the voluntary cough motor imagery practice group is summarized in Table 6 and Fig. 4. Significant increases in PEF from cough spirometry (+ 1.00, 95% CI: 0.12, 1.9, *p* = *0.04*) were observed for the voluntary cough motor imagery practice group. No other statistically significant increases were observed across the voluntary cough motor imagery practice group.

**Table 6:** Summary of the median and interquartile range (IQR) of pulmonary and cough function outcome measures during baseline and post-motor imagery practice for the voluntary cough (VC) group

Outcome measures	Median (IQR) Baseline 1	Median (IQR) Baseline 2	Median (IQR) Post-Voluntary Cough MP	Mean Difference (95% CI)	P-value
Pulmonary function tests					
Maximum expiratory pressure (cm H20)	127 (117, 149)	138 (122, 166)	125 (119, 177)	− 0.8 (− 11.9, 10.3)	0.42
Maximum inspiratory pressure (cm H20)	86 (71, 107)	97 (79, 110)	98 (78, 112)	2.4 (− 3.3, 8.1)	0.41
Forced vital capacity (% predicted)	110 (90, 141)	112 (94, 138)	110 (92, 134)	− 9.3 (− 22.7, 4.1)	0.02*
Voluntary cough peak expiratory flow and cough spirometry metrics					
Peak expiratory flow rate from a handheld device (L/min)	413 (365, 473)	435 (414, 469)	450 (420, 465)	12 (− 6.4, 30.4)	0.19
Inspiratory phase duration (s)	1.6 (1.1, 2.4)	1.5 (1.1, 2.2)	1.7 (1.3, 2.7)	0.4 (− 0.5, 1.3)	0.43
Inspiratory peak flow rate (L/s)	− 1.9 (− 2.2, − 1.4)	− 1.9 (− 3.1, − 1.2)	− 1.9 (− 4.1, − 1.6)	− 0.45 (− 1.1, 0.17)	0.23
Compression phase duration (s)	0.2 (0.1, 0.2)	0.1 (0.1, 0.2)	0.1 (0.1, 0.2)	− 0.02 (− 0.2, 0.2)	0.56
Expiratory phase duration (s)	0.04 (0.03, 0.04)	0.04 (0.03, 0.04)	0.04 (0.03, 0.04)	0.001 (− 0.008, 0.01)	0.46
Peak expiratory flow rate from cough spirometry (L/s)	7.7 (7.3, 8.0)	7.6 (6.9, 9.2)	8.8 (7.5, 9.5)	1.0 (0.1, 1.9)	0.04*
Cough volume acceleration (L/s/s)	228.8 (168.9, 239.5)	232.3 (159.4, 255.5)	242.2 (214.1, 292.7)	29.8 (− 15.9, 75.7)	0.19

### Individual Variability Performance Data Within Sessions and Across Timepoints

A complete summary of individual variability within sessions and across sessions for all pulmonary and cough function outcome measures is provided in the Supplementary Materials.

## Discussion

This prospective study found that motor imagery practice of EMST and voluntary cough was feasible, minimally burdensome, and led to statistically significant improvements in voluntary PEF using a handheld peak cough flow meter and one voluntary cough spirometry metric (PEF) in a small cohort of young community-dwelling adults. Statistically significant improvements were not observed for MEP, MIP, FVC, or any additional voluntary cough spirometry metrics. These findings are encouraging given that healthy adults may experience ceiling effects even in active treatment studies. Future large-scale clinical trials in patient populations with pulmonary, cough, and/or swallowing impairments may yield greater improvements across all pulmonary and cough function outcome measures.

Using a phone application for logging motor imagery practice repetitions (MyCap) and a convenient at home treatment program with weekly telehealth sessions likely contributed to the high adherence, treatment session attendance, and minimal treatment burden reported by research participants. This demonstrates the high potential to translate this treatment program to clinical populations.

In line with prior active voluntary cough and EMST treatment studies [15–31], we observed improvements in voluntary PEF following motor imagery practice. This finding provides encouraging evidence that motor imagery practice of EMST and voluntary cough may be a useful treatment for patients given that improving cough effectiveness can yield to improve swallowing safety in individuals with dysphagia. Surprisingly, motor imagery practice of EMST did not lead to statistically significant improvements in MEP, which is typically the primary outcome measure of active EMST clinical trials. Perhaps this lack of improvement in the EMST motor imagery practice is due to increased difficulty visualizing the EMST motor imagery practice task compared to the voluntary cough task given that coughing is a familiar motor pattern. While we did not observe improvements in MIP, FVC, or other voluntary cough spirometry metrics, we would not anticipate generalization to these breathing and cough function measures based on the motor imagery practice repetitions prescribed in this study.

Similar to prior studies examining motor imagery practice of tongue strengthening exercises or the limbs over six weeks, we used a five-week training protocol to examine the feasibility and efficacy of motor imagery practice of EMST and voluntary cough. We observed a percent change ranging from 2–16% in PEF from the baseline two to post-treatment timepoints. This percent change is slightly reduced compared to Szynkiewicz et al. (2019), which found a mean percent change of 26% in tongue strength from baseline to 6 weeks post-treatment [7]. The larger improvement observed post-treatment from the Szynkiewicz et al. (2019) study may be due to methodological differences in study design. In this previous study, maximum isometric tongue pressure measurements were obtained from participants at baseline, 2 weeks, 4 weeks, and 6 weeks. Furthermore, participants had an additional in person training session two days after the initial training to ensure retention and accuracy of repetitions. The training protocol also differed from the current study and consisted of 30 repetitions, three times per day, three days per week. Lastly, this prior study incorporated a progressive imagined resistance load from 60–80% over the 6-week training period, while the current study had a consistent imagined training load of 70%. In contrast to this, a randomized controlled trial in 29 healthy older adults that compared active completion of lingual exercises, motor imagery practice of lingual exercises, a combined intervention group, and a control group found that motor imagery practice alone led to only 6% improvement in tongue strength post-treatment, while the combined active/motor imagery practice group led to 17% improvement in tongue-strength post-treatment [13]. These noted improvements are more in line with the present study which found 2–16% improvements in PEF post-treatment.

### Study Limitations

While the preliminary findings from this study are promising, there are several important limitations to acknowledge. Given the pilot nature of this study, the sample size was relatively small (n = 20), most enrolled participants were female (84%), and the age of participants was relatively young (33.8 ± 10.4), which may have resulted in a ceiling effect. Additionally, due to the exploratory nature of this study, we did not control for multiple comparisons because we primarily aimed to assess feasibility and trends of treatment efficacy for motor imagery practice of voluntary cough and EMST. Future motor imagery practice research is needed in larger, more diverse (e.g., sex, age, race, etc.) samples of community-dwelling adults in order to draw firm conclusions regarding treatment efficacy and to allow for more robust performance of statistical analysis. This study focused on the feasibility and impact of motor imagery practice of EMST and voluntary cough in a cohort of community-dwelling adults. It will be important for future research to examine the feasibility and impact of motor imagery practice of EMST and voluntary cough in individuals with pulmonary, cough, and/or swallowing impairments. To minimize participant burden and ensure feasibility for this study, weekly treatment check-ins were conducted via telehealth. Ideally, weekly visits would be conducted in person each week to ensure accuracy of motor imagery practice and to reassess pulmonary and cough function measures. Additionally, while we utilized a multiple baseline design to account for a learning effect in this study, we did not formally control for a learning effect beyond this given the relatively small sample size, preliminary nature of this study, and primary goal of examining feasibility and trends of treatment efficacy. Notably, some individuals had high test–retest reliability across sessions one and two, while other individuals demonstrated greater variability in performance which may have impacted the overall study results. Performance variability across testing days/times in healthy adults and patient populations may warrant additional exploration. Future studies may also consider incorporating more in-person visits throughout treatment to ensure treatment fidelity. Given that the participants enrolled in this study were healthy community-dwelling adults, we did not perform instrumental swallowing assessments during research evaluations. In the future, it will be important to assess swallow function using imaging pre- and post-motor imagery practice of EMST and voluntary cough when determining the impact on swallow function in patient populations with co-occurring impairments in pulmonary, cough, and/or swallow function. Lastly, this study did not perform fMRI during active performance of voluntary cough and EMST or during motor imagery practice of voluntary cough and EMST to ensure accuracy of motor imagery repetitions. While participants were monitored for overt movements or muscular activation of the head, neck, and trunk muscles during treatment sessions and were cognitively intact/aware they were not to perform any active movements, it is possible that some muscle activation occurred during sessions. However, given that no active resistance was present, it is unlikely that any significant gains in muscle strength/performance on pulmonary or cough tasks would be due to these small motor movements rather than motor imagery practice. In the future, it would be beneficial to obtain baseline and post-treatment neuroimaging during active performance and motor imagery practice performance of voluntary cough and EMST to determine brain activation patterns between these conditions.

## Conclusions

Preliminary findings suggest that motor imagery practice of voluntary cough and EMST is feasible and may yield improvements in voluntary cough function in community-dwelling adults. Future research work is necessary in larger, more diverse sample sizes of community dwelling-adults and in patient populations with pulmonary, cough, and/or swallowing impairments to confirm and expand upon these findings to determine the utility of motor imagery practice as a potential adjuvant dysphagia treatment.

## Supplementary Information

Below is the link to the electronic supplementary material.

Supplementary file1 (PDF 943 kb)

## Data Availability

Deidentified data available upon reasonable request.
