# Supplementary material for: Motor Imagery Practice to Improve Respiratory and Cough Function
Source: Dysphagia. 2025 Feb 26;40(5):1214–27. doi: 10.1007/s00455-025-10818-2 (PMC12479617; doi:10.1007/s00455-025-10818-2)
Supplement: Supplementary file 1 — Supplementary file1 (PDF 943 kb) [file 455_2025_10818_MOESM1_ESM.pdf]

Supplementary Materials:

1) Individual research participant graphs demonstrating individual variability across three maximum expiratory pressure (MEP) measures (within each session) and across time points (baseline 1, baseline 2, post-treatment) for the MP EMST group. Across each timepoint, the median is shown with error bars indicating minimum and maximum values.

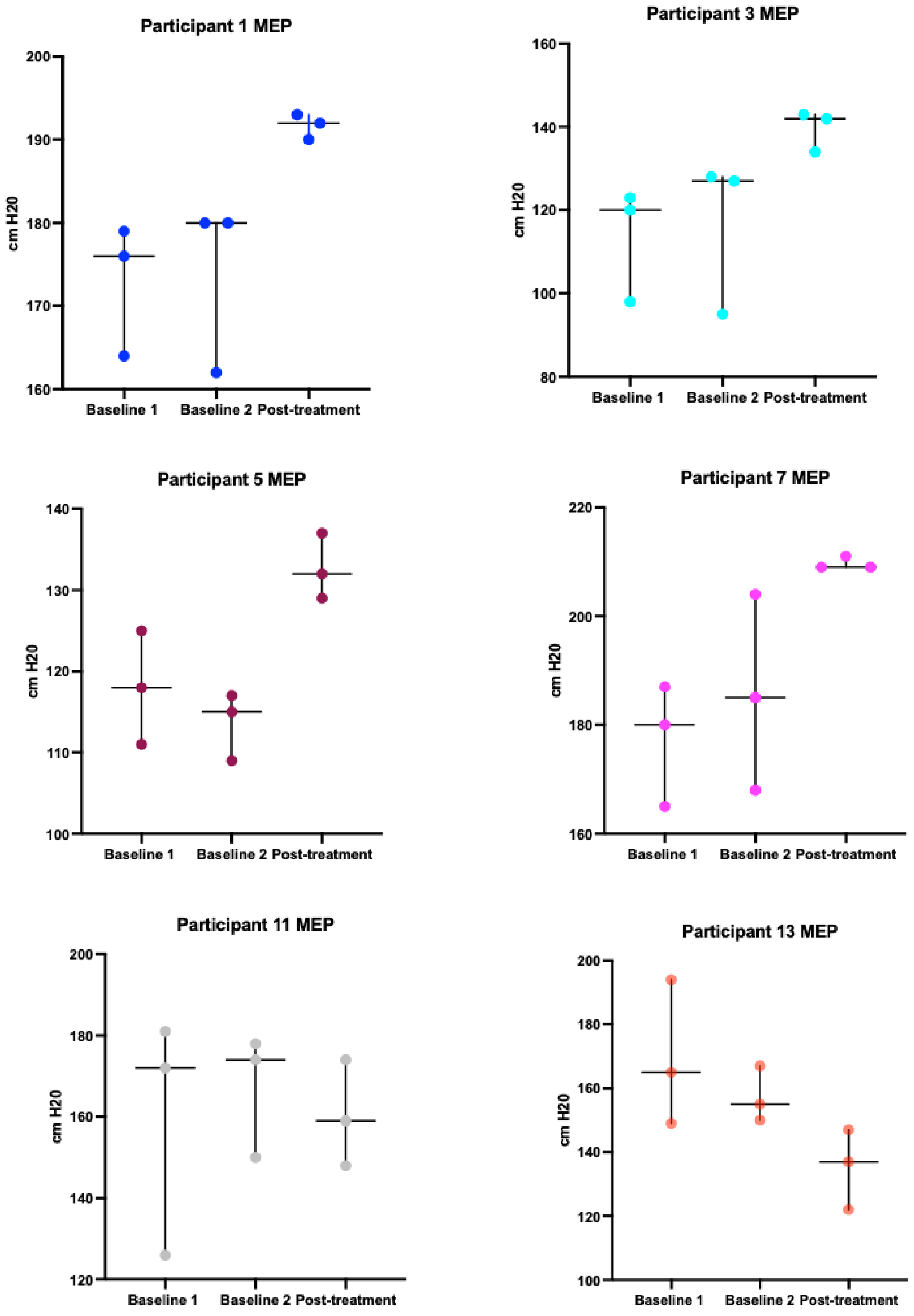

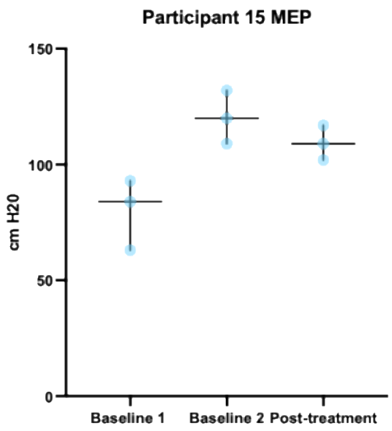

Participant 15 MEP
150
100
50
0
cm H20
Baseline 1
Baseline 2
Post-treatment

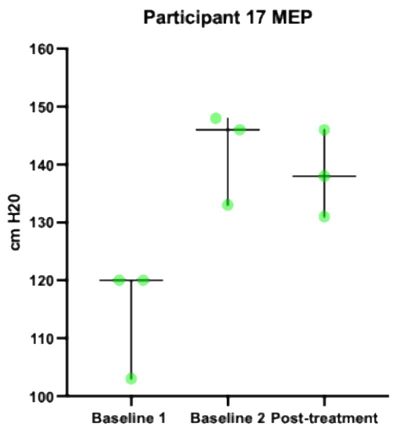

Participant 17 MEP
160
150
140
130
120
110
100
cm H20
Baseline 1
Baseline 2
Post-treatment

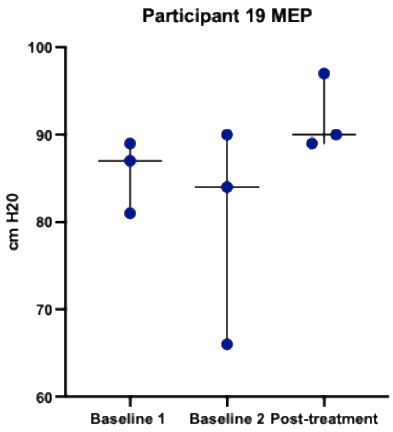

Participant 19 MEP
100
90
80
70
60
cm H20
Baseline 1
Baseline 2
Post-treatment

2) Individual research participant graphs demonstrating individual variability across three maximum inspiratory pressure (MIP) measures (within each session) and across time points (baseline 1, baseline 2, post-treatment) for the MP EMST group. Across each timepoint, the median is shown with error bars indicating minimum and maximum values.

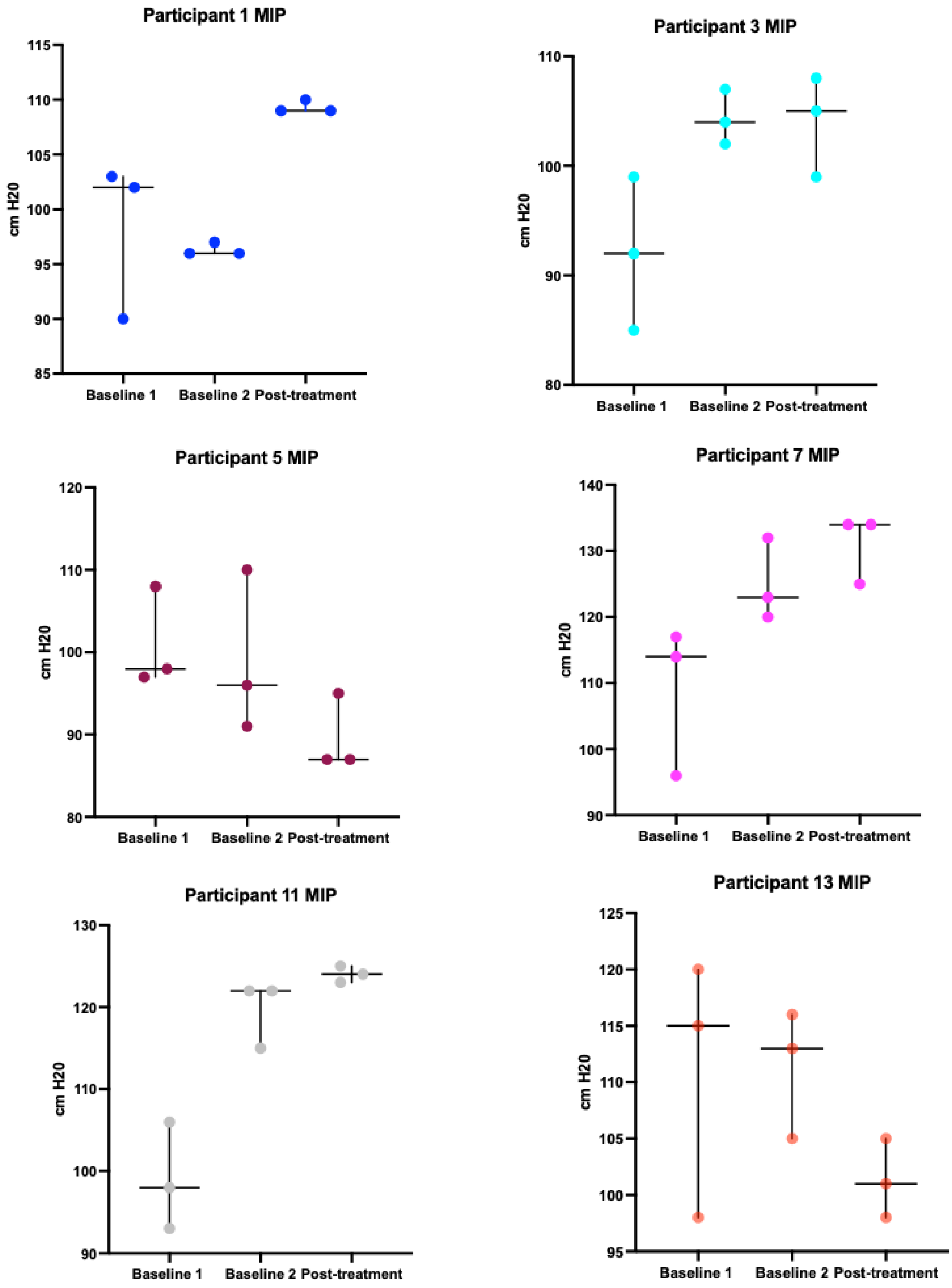

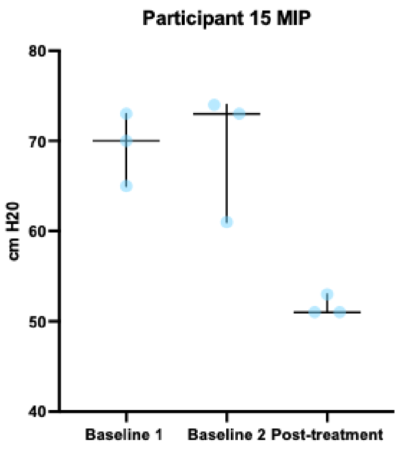
Participant 15 MIP
80
70
60
50
40
cm H20
Baseline 1
Baseline 2
Post-treatment

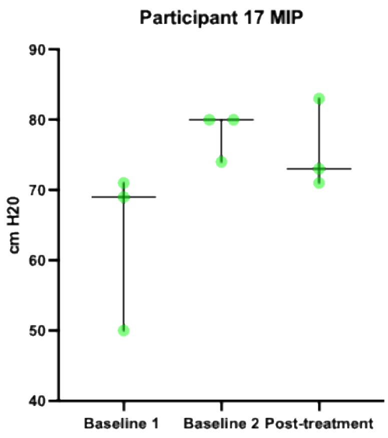
Participant 17 MIP
90
80
70
60
50
40
cm H20
Baseline 1
Baseline 2
Post-treatment

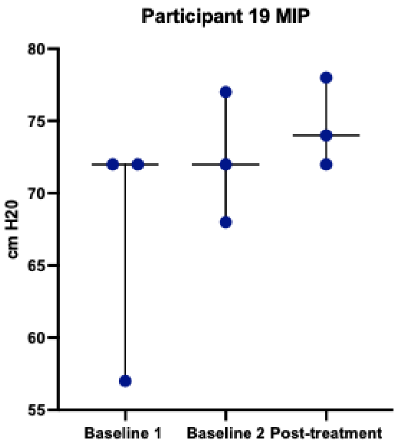
Participant 19 MIP
80
75
70
65
60
55
cm H20
Baseline 1
Baseline 2
Post-treatment

3) Individual research participant graphs demonstrating individual variability across three forced vital capacity (FVC) measures (within each session) and across time points (baseline 1, baseline 2, post-treatment) for the MP EMST group. Across each timepoint, the median is shown with error bars indicating minimum and maximum values.

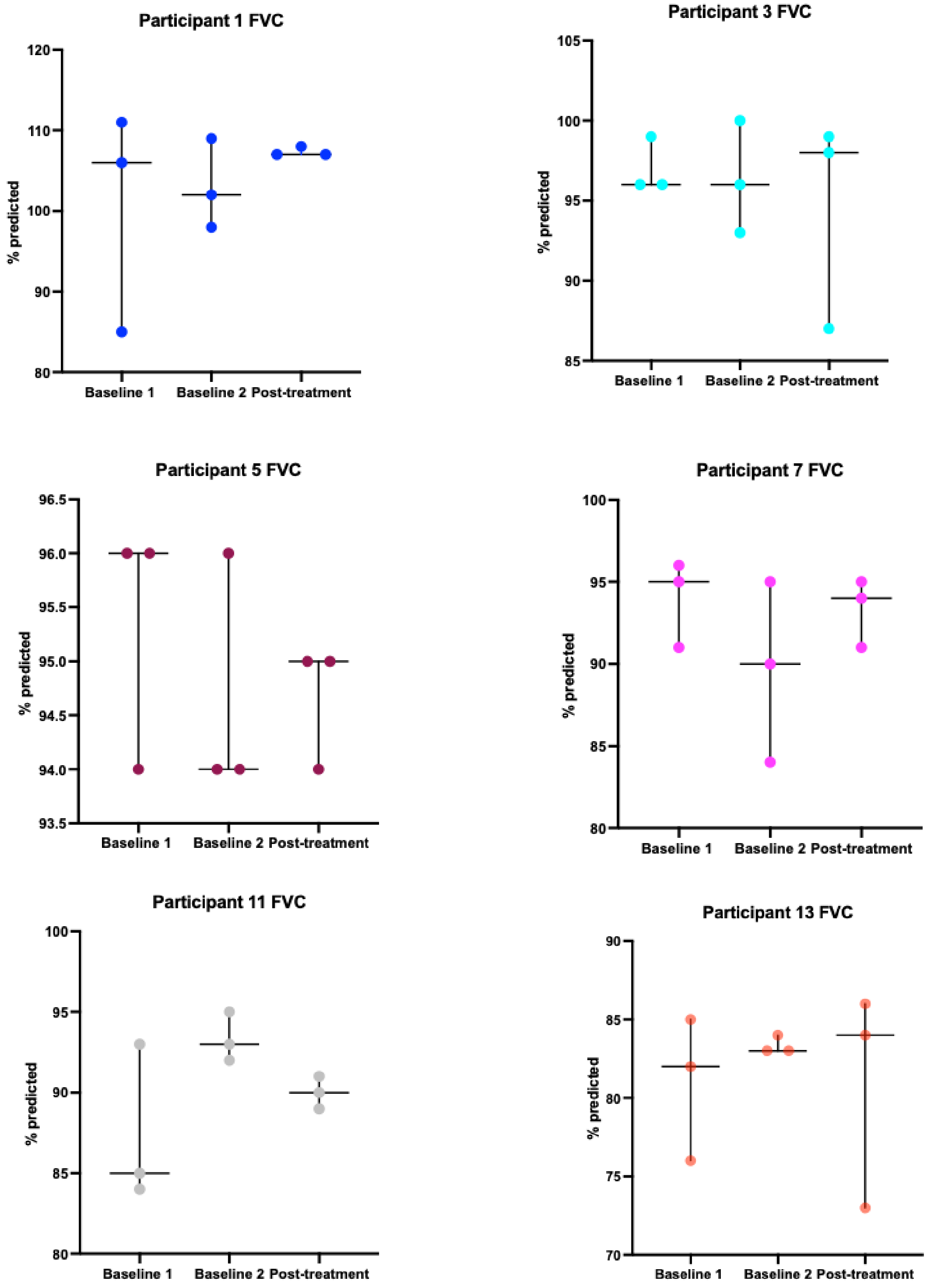

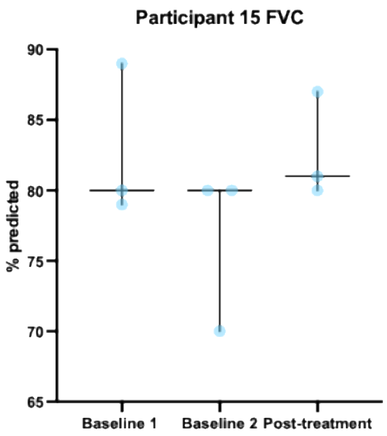
Participant 15 FVC
% predicted
90
85
80
75
70
65
Baseline 1
Baseline 2
Post-treatment

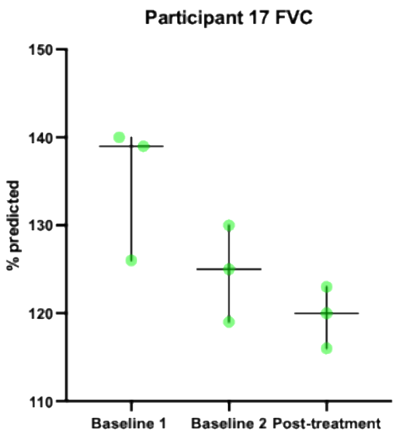
Participant 17 FVC
% predicted
150
140
130
120
110
Baseline 1
Baseline 2
Post-treatment

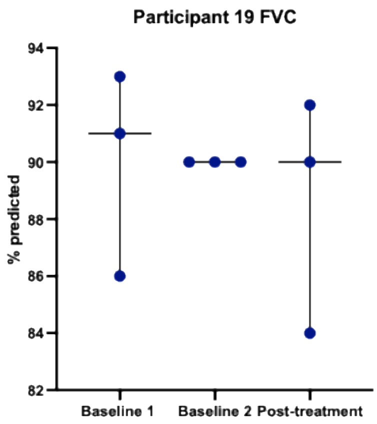
Participant 19 FVC
% predicted
94
92
90
88
86
84
82
Baseline 1
Baseline 2
Post-treatment

4) Individual research participant graphs demonstrating individual variability across three voluntary cough peak expiratory flow (PCF) measures (within each session) and across time points (baseline 1, baseline 2, post-treatment) for the MP EMST group. Across each timepoint, the median is shown with error bars indicating minimum and maximum values.

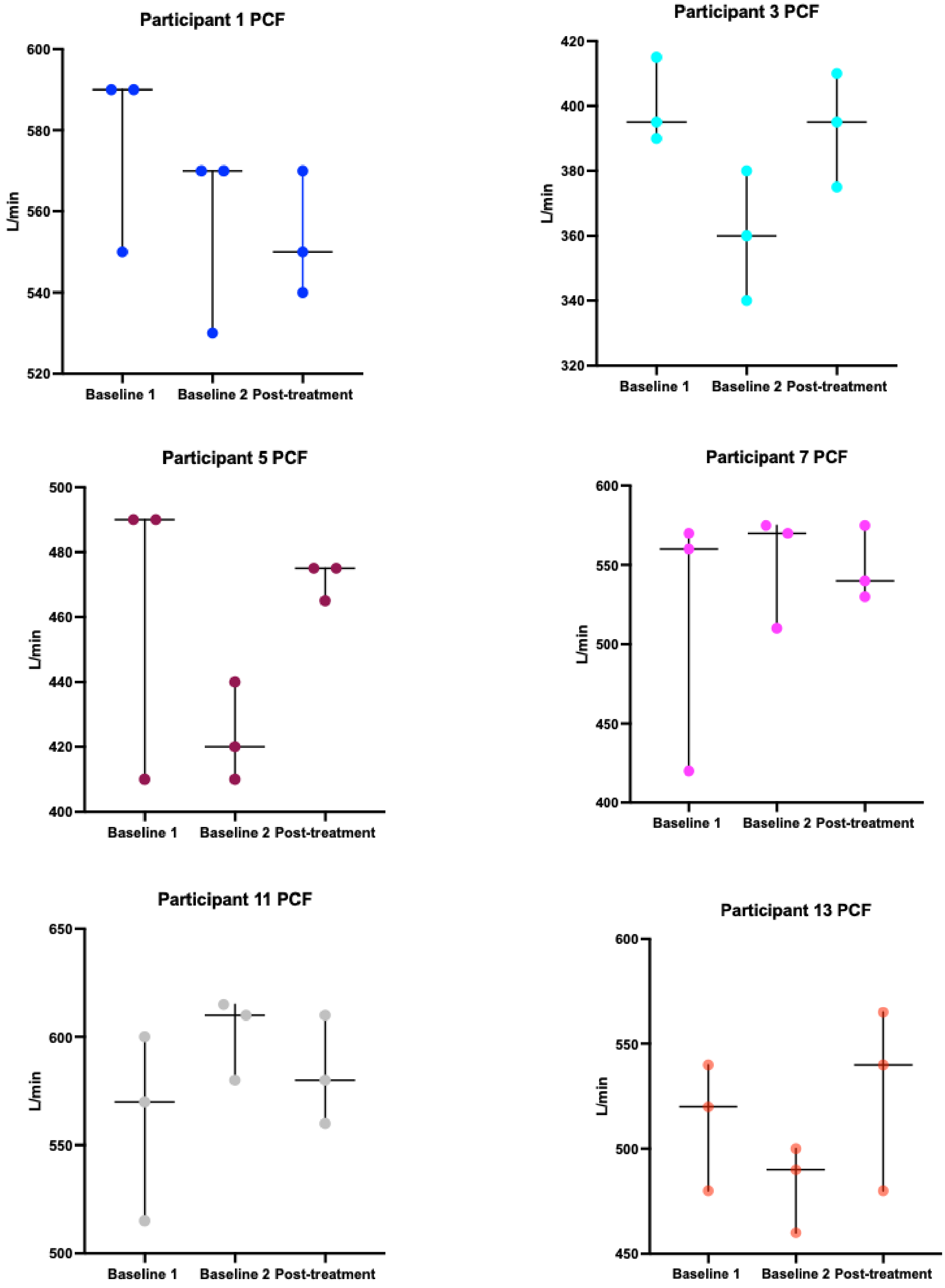

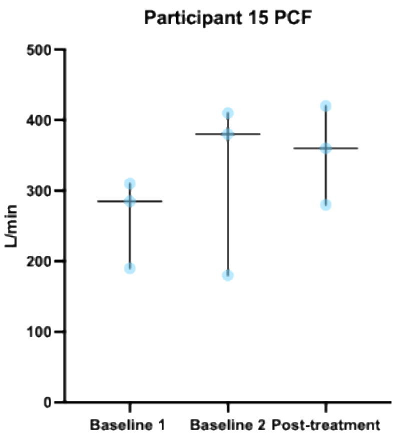
Participant 15 PCF
500
400
300
200
100
0
L/min
Baseline 1
Baseline 2
Post-treatment

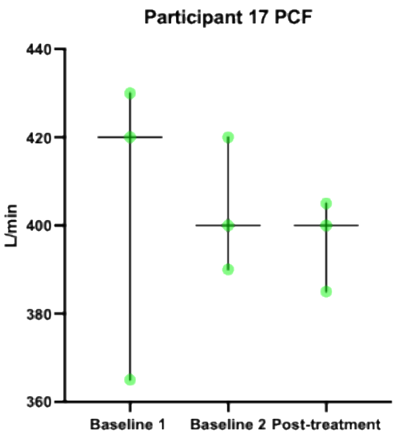
Participant 17 PCF
440
420
400
380
360
L/min
Baseline 1
Baseline 2
Post-treatment

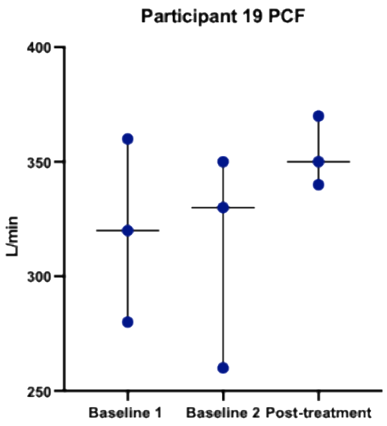
Participant 19 PCF
400
350
300
250
L/min
Baseline 1
Baseline 2
Post-treatment

5) Individual research participant graphs demonstrating individual variability across three peak expiratory flow rate measures (within each session) and across time points (baseline 1, baseline 2, post-treatment) for the MP EMST group. Across each timepoint, the median is shown with error bars indicating minimum and maximum values.

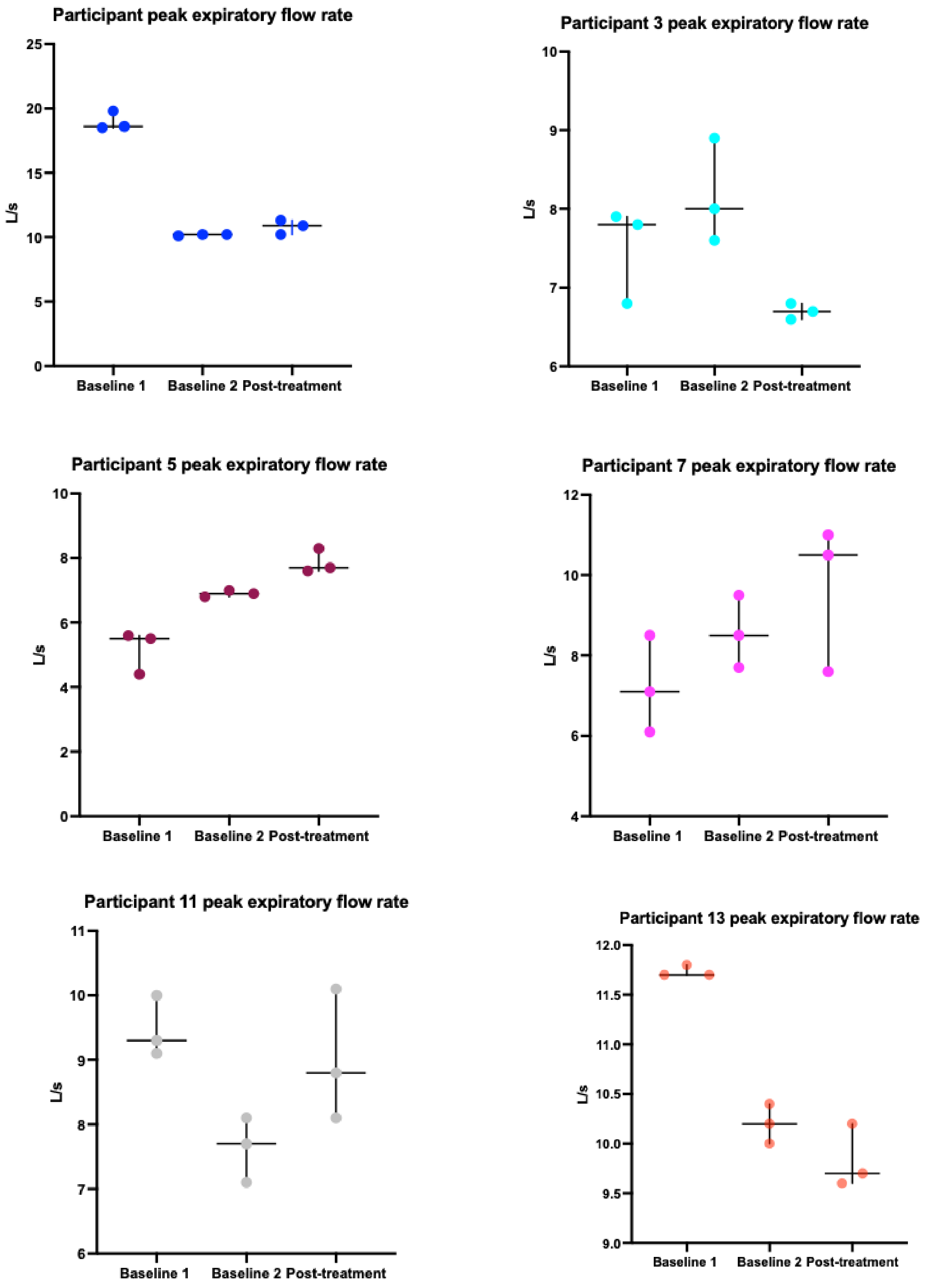

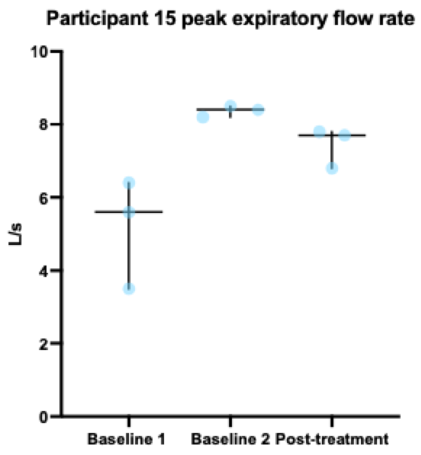
Participant 15 peak expiratory flow rate
10
8
6
4
2
0
L/s
Baseline 1
Baseline 2
Post-treatment

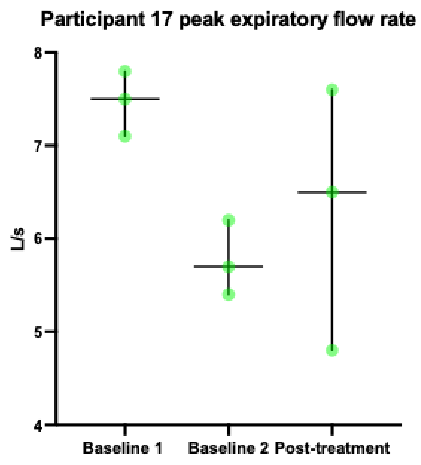
Participant 17 peak expiratory flow rate
8
7
6
5
4
L/s
Baseline 1
Baseline 2
Post-treatment

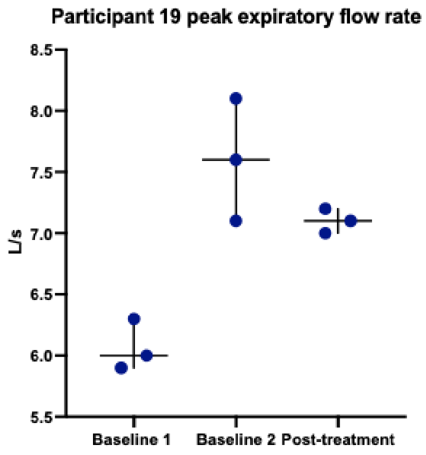
Participant 19 peak expiratory flow rate
8.5
8.0
7.5
7.0
6.5
6.0
5.5
L/s
Baseline 1
Baseline 2
Post-treatment

1) Individual research participant graphs demonstrating individual variability across three maximum expiratory pressure (MEP) measures (within each session) and across time points (baseline 1, baseline 2, post-treatment) for the MP VC group. Across each timepoint, the median is shown with error bars indicating minimum and maximum values.

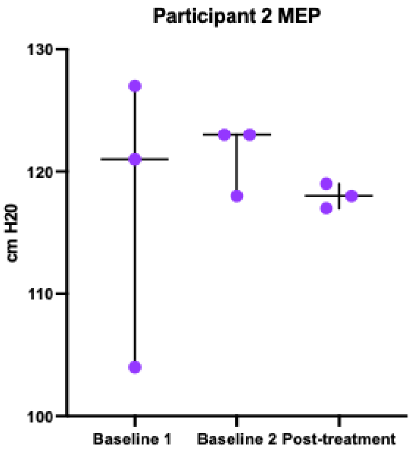

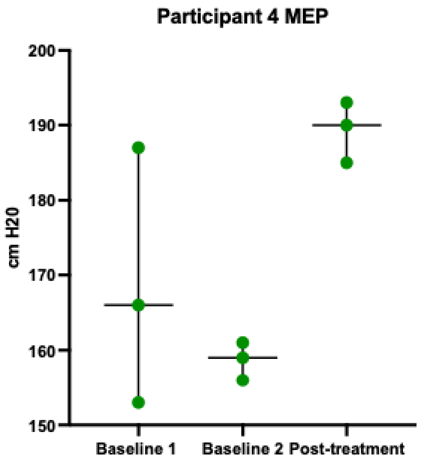

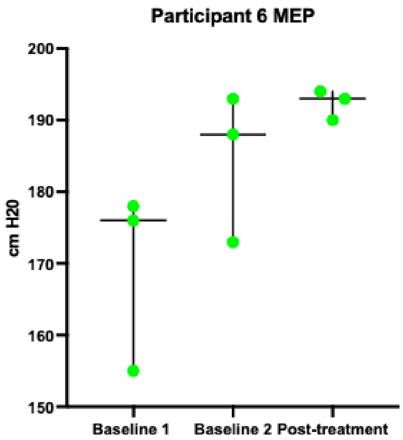

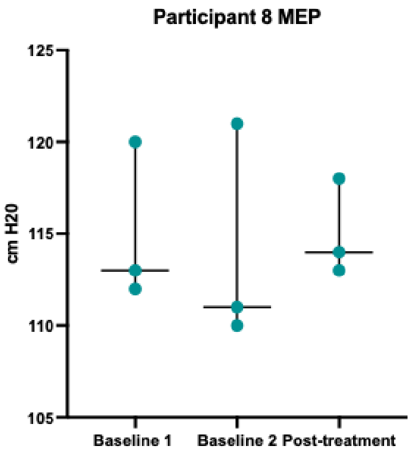

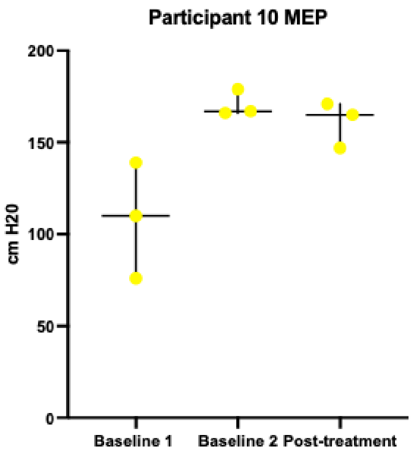

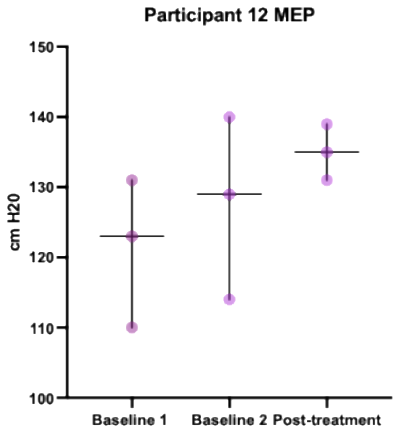

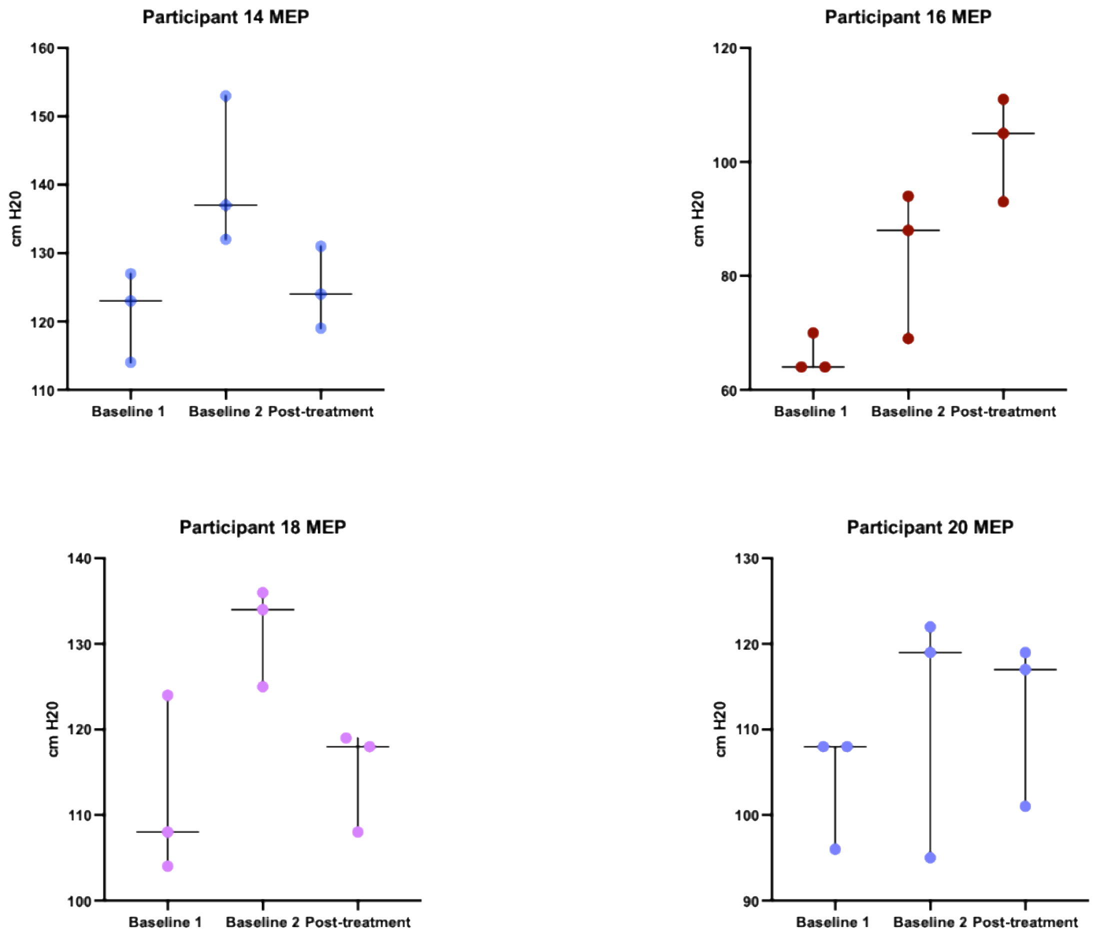
Participant 14 MEP
cm H20
160
150
140
130
120
110
Baseline 1
Baseline 2
Post-treatment
Participant 16 MEP
cm H20
120
100
80
60
Baseline 1
Baseline 2
Post-treatment
Participant 18 MEP
cm H20
140
130
120
110
100
Baseline 1
Baseline 2
Post-treatment
Participant 20 MEP
cm H20
130
120
110
100
90
Baseline 1
Baseline 2
Post-treatment

2) Individual research participant graphs demonstrating individual variability across three maximum inspiratory pressure (MIP) measures (within each session) and across time points (baseline 1, baseline 2, post-treatment) for the MP VC group. Across each timepoint, the median is shown with error bars indicating minimum and maximum values.

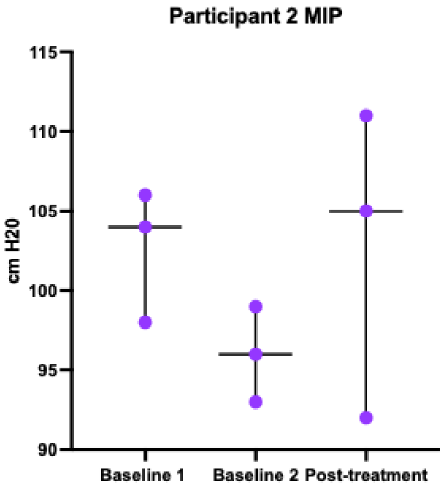

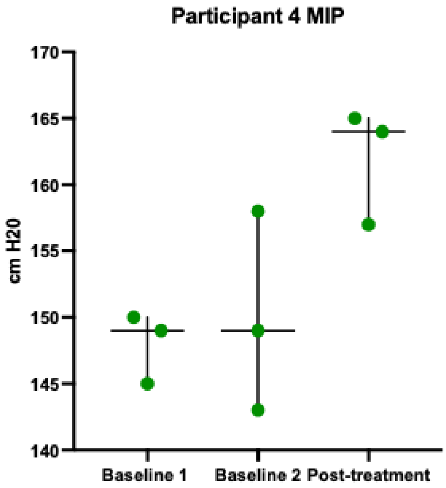

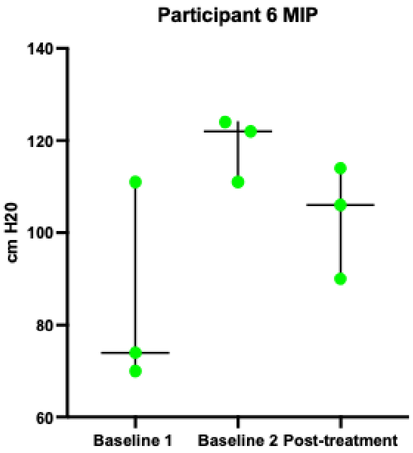

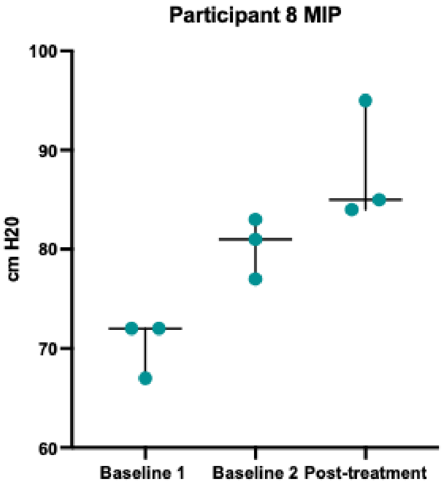

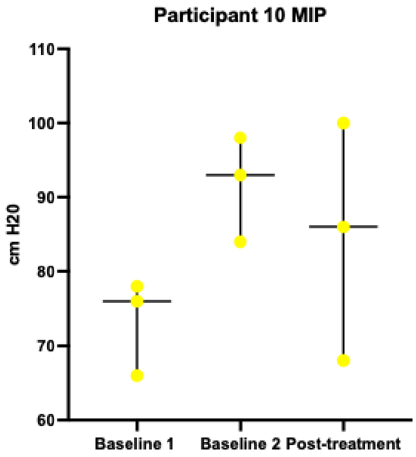

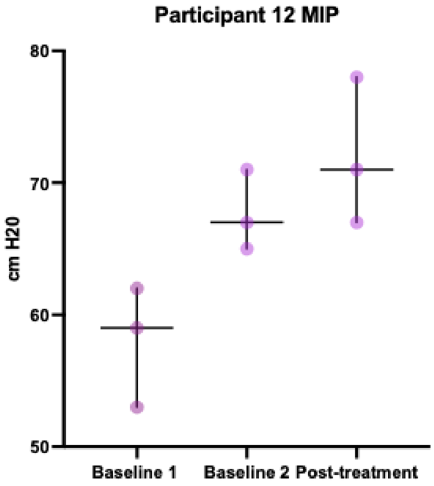

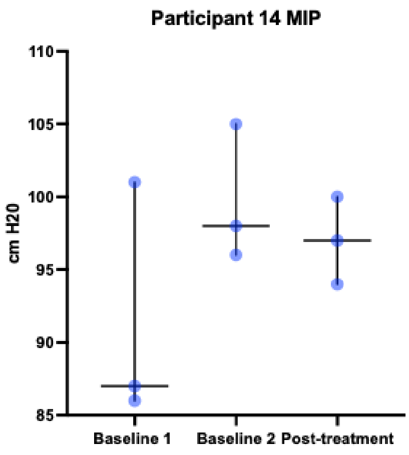
Participant 14 MIP
cm H20
110
105
100
95
90
85
Baseline 1
Baseline 2
Post-treatment

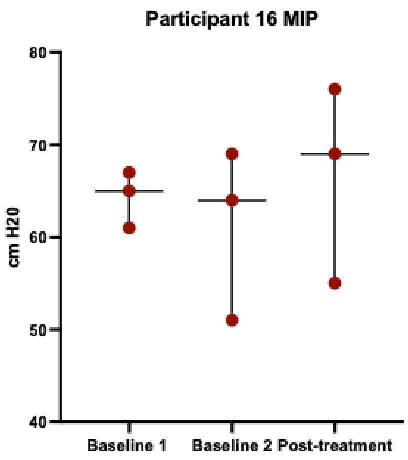
Participant 16 MIP
cm H20
80
70
60
50
40
Baseline 1
Baseline 2
Post-treatment

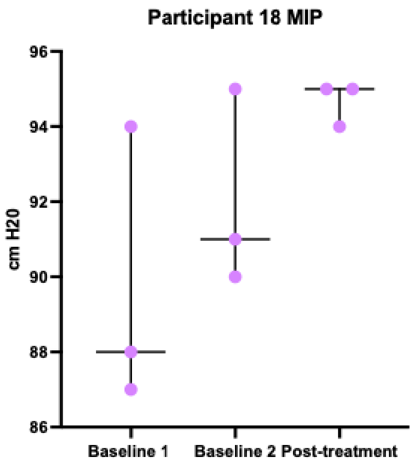
Participant 18 MIP
cm H20
96
94
92
90
88
86
Baseline 1
Baseline 2
Post-treatment

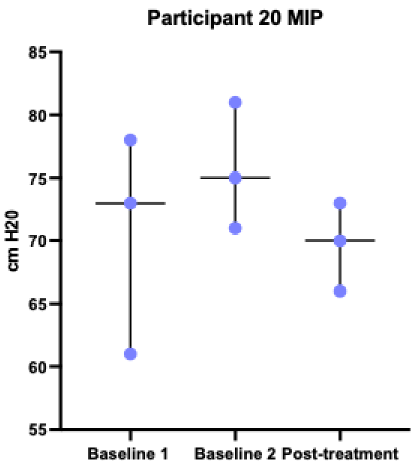
Participant 20 MIP
cm H20
85
80
75
70
65
60
55
Baseline 1
Baseline 2
Post-treatment

3) Individual research participant graphs demonstrating individual variability across three forced vital capacity (FVC) measures (within each session) and across time points (baseline 1, baseline 2, post-treatment) for the MP VC group. Across each timepoint, the median is shown with error bars indicating minimum and maximum values.

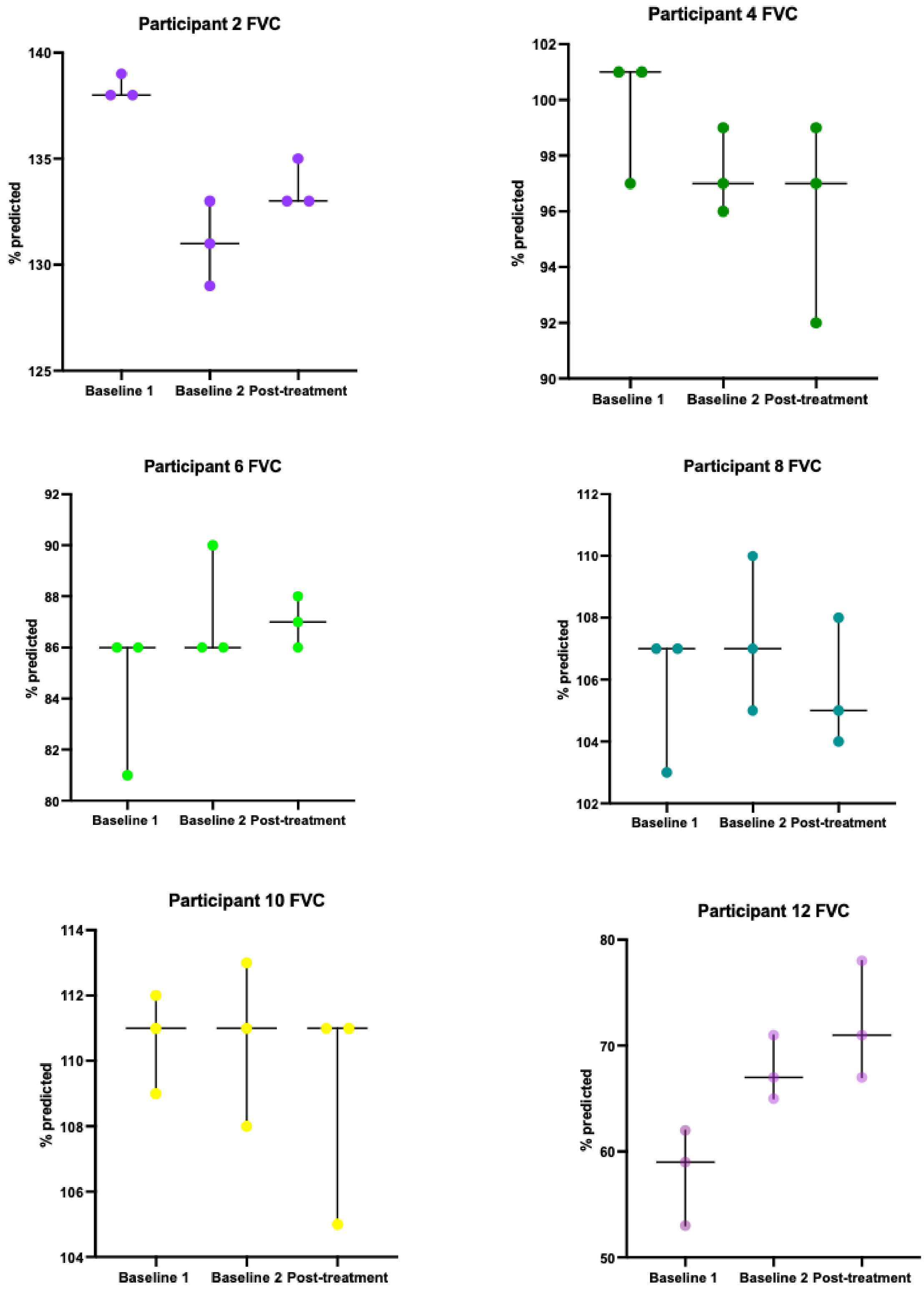

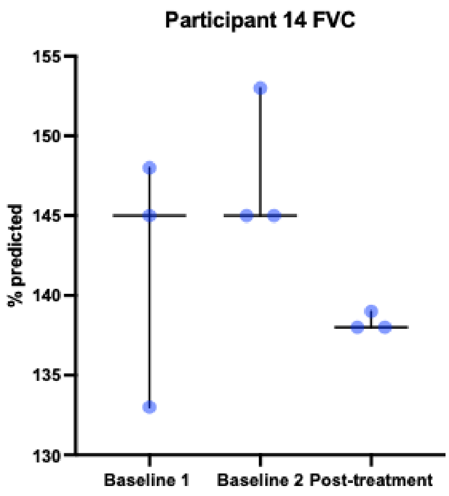
Participant 14 FVC
% predicted
155
150
145
140
135
130
Baseline 1
Baseline 2
Post-treatment

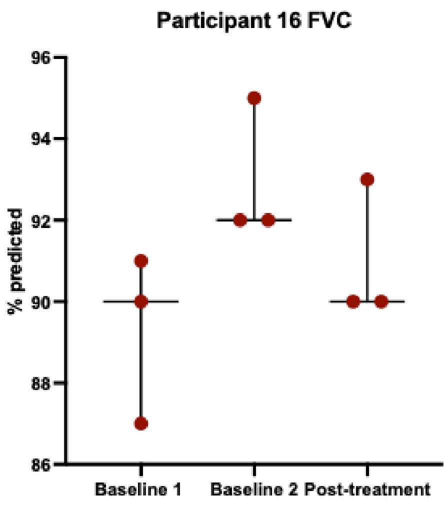
Participant 16 FVC
% predicted
96
94
92
90
88
86
Baseline 1
Baseline 2
Post-treatment

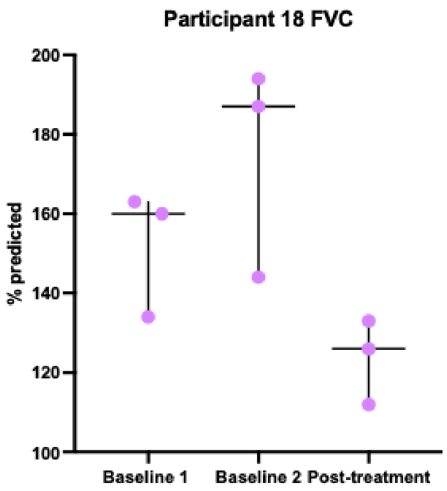
Participant 18 FVC
% predicted
200
180
160
140
120
100
Baseline 1
Baseline 2
Post-treatment

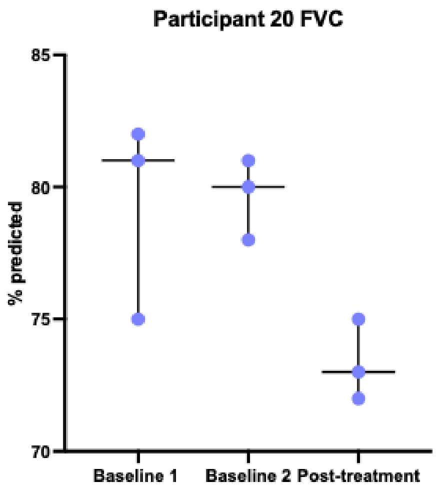
Participant 20 FVC
% predicted
85
80
75
70
Baseline 1
Baseline 2
Post-treatment

4) Individual research participant graphs demonstrating individual variability across three voluntary cough peak expiratory flow (PCF) measures (within each session) and across time points (baseline 1, baseline 2, post-treatment) for the MP VC group. Across each timepoint, the median is shown with error bars indicating minimum and maximum values.

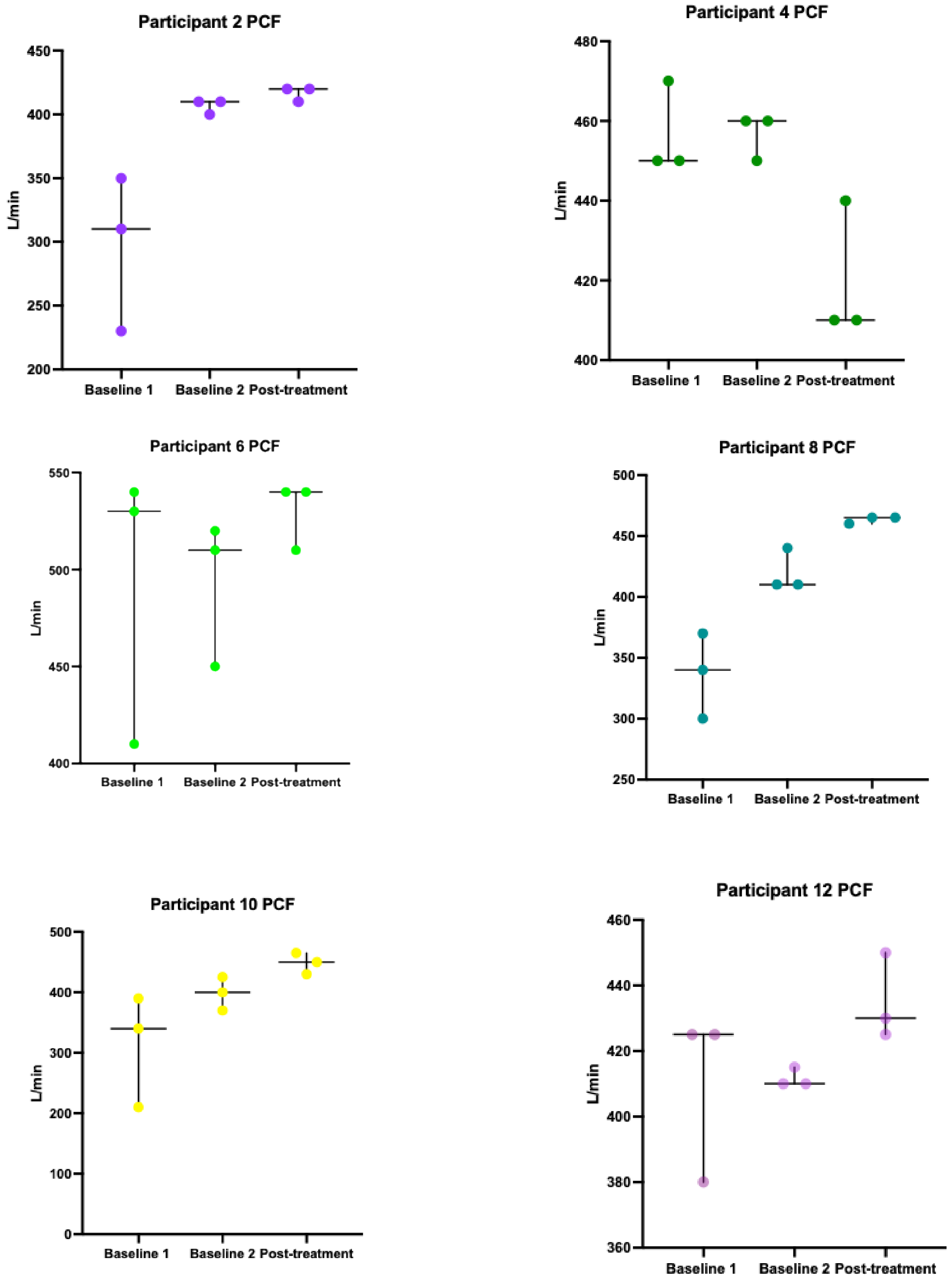

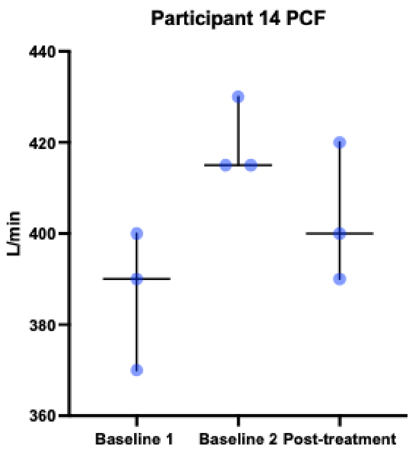
Participant 14 PCF
L/min
440
420
400
380
360
Baseline 1
Baseline 2
Post-treatment

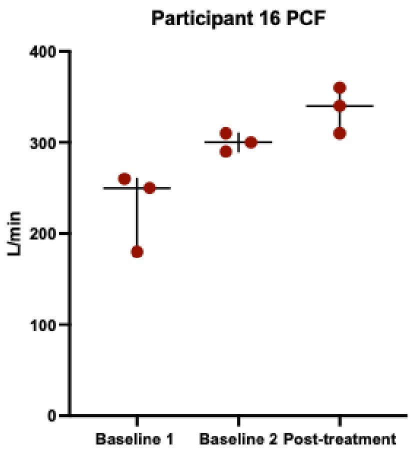
Participant 16 PCF
L/min
400
300
200
100
0
Baseline 1
Baseline 2
Post-treatment

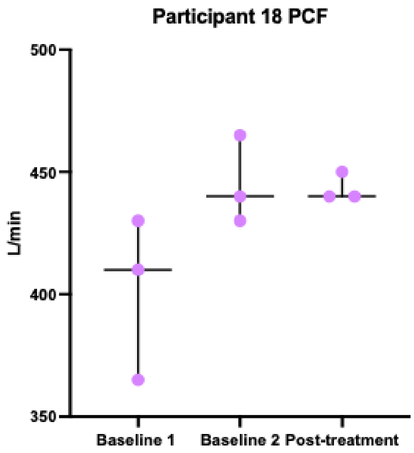
Participant 18 PCF
L/min
500
450
400
350
Baseline 1
Baseline 2
Post-treatment

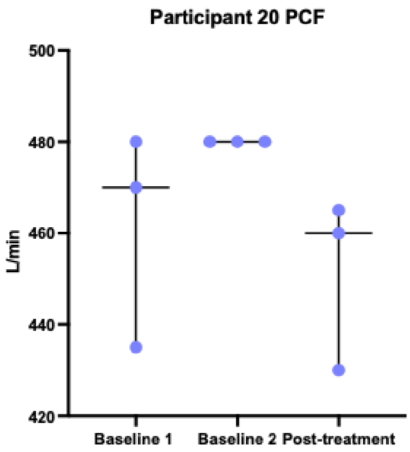
Participant 20 PCF
L/min
500
480
460
440
420
Baseline 1
Baseline 2
Post-treatment

5) Individual research participant graphs demonstrating individual variability across three peak expiratory flow rate measures (within each session) and across time points (baseline 1, baseline 2, post-treatment) for the MP VC group. Across each timepoint, the median is shown with error bars indicating minimum and maximum values.

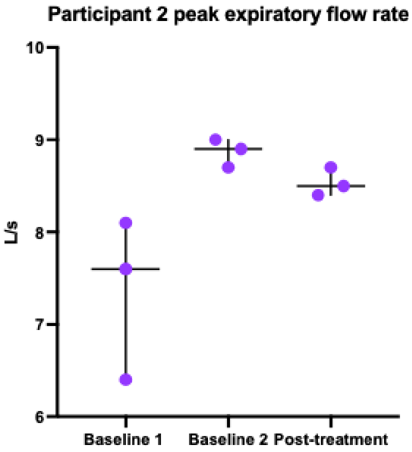

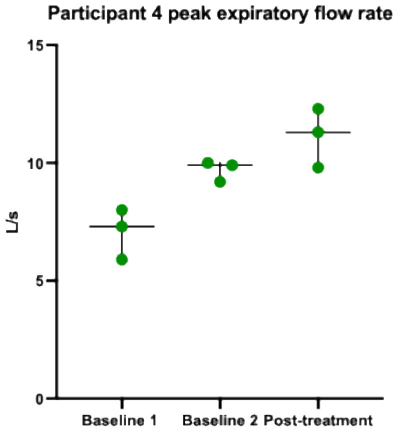

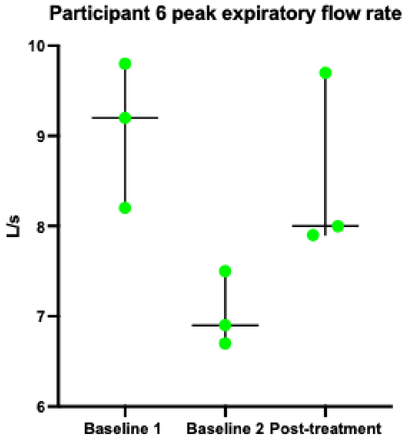

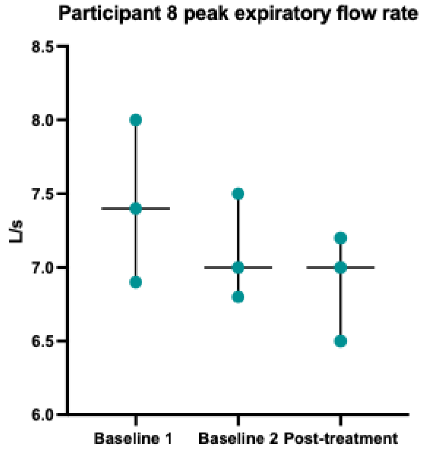

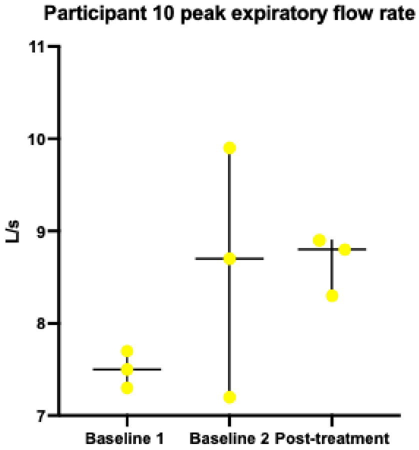

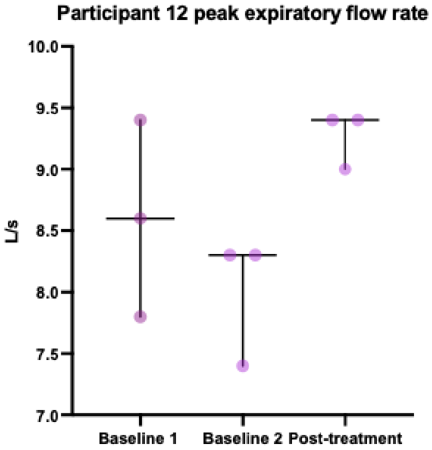

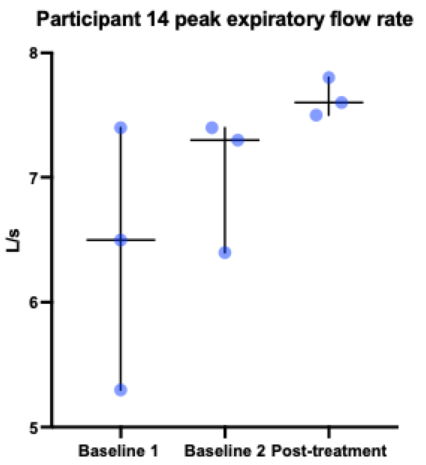
Participant 14 peak expiratory flow rate
L/s
8
7
6
5
Baseline 1
Baseline 2
Post-treatment

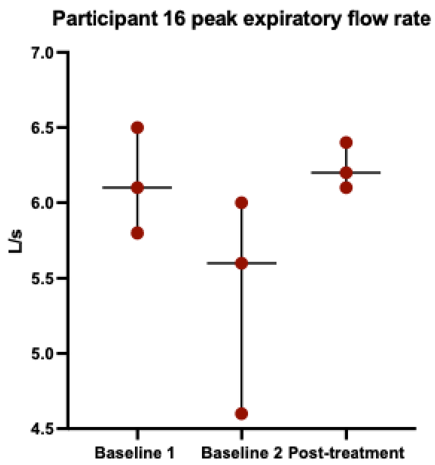
Participant 16 peak expiratory flow rate
L/s
7.0
6.5
6.0
5.5
5.0
4.5
Baseline 1
Baseline 2
Post-treatment

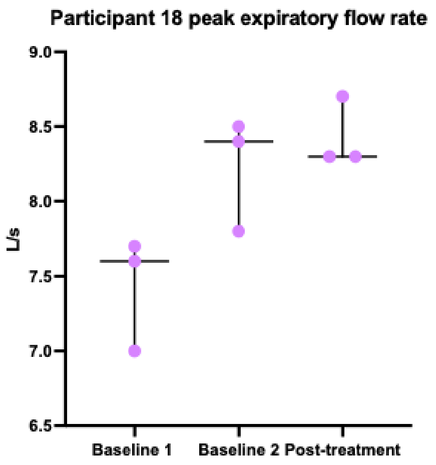
Participant 18 peak expiratory flow rate
L/s
9.0
8.5
8.0
7.5
7.0
6.5
Baseline 1
Baseline 2
Post-treatment

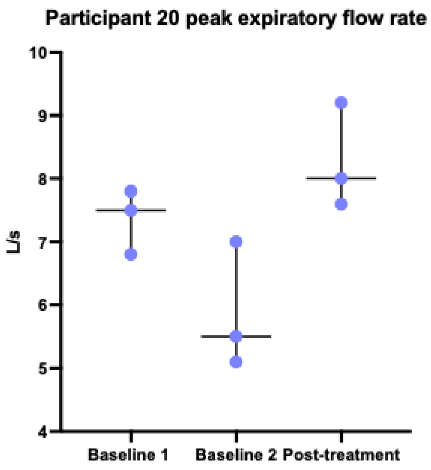
Participant 20 peak expiratory flow rate
L/s
10
9
8
7
6
5
4
Baseline 1
Baseline 2
Post-treatment
